# Distinct spatial arrangements of ACE2 and TMPRSS2 expression in Syrian hamster lung lobes dictates SARS-CoV-2 infection patterns

**DOI:** 10.1371/journal.ppat.1010340

**Published:** 2022-03-07

**Authors:** Ilhan Tomris, Kim M. Bouwman, Youri Adolfs, Danny Noack, Roosmarijn van der Woude, Gius Kerster, Sander Herfst, Rogier W. Sanders, Marit J. van Gils, Geert-Jan Boons, Bart L. Haagmans, R. Jeroen Pasterkamp, Barry Rockx, Robert P. de Vries

**Affiliations:** 1 Department of Chemical Biology & Drug Discovery, Utrecht Institute for Pharmaceutical Sciences, Utrecht University, Utrecht, The Netherlands; 2 Department of Translational Neuroscience, University Medical Center Utrecht Brain Center, Utrecht University, Utrecht, The Netherlands; 3 Department of Viroscience, Erasmus University Medical Center, Rotterdam, The Netherlands; 4 Department of Medical Microbiology, Amsterdam UMC, University of Amsterdam, Amsterdam Institute for Infection and Immunity, Amsterdam, The Netherlands; 5 Department of Microbiology and Immunology, Weill Medical College of Cornell University, Ney York City, New York, United States of America; 6 Bijvoet Center for Biomolecular Research, Utrecht University, Utrecht, The Netherlands; 7 Complex Carbohydrate Research Center, University of Georgia, Athens, Georgia, United States of America; 8 Department of Chemistry, University of Georgia, Athens, Georgia, United States of America; University of Pittsburgh, UNITED STATES

## Abstract

SARS-CoV-2 attaches to angiotensin-converting enzyme 2 (ACE2) to gain entry into cells after which the spike protein is cleaved by the transmembrane serine protease 2 (TMPRSS2) to facilitate viral-host membrane fusion. ACE2 and TMPRSS2 expression profiles have been analyzed at the genomic, transcriptomic, and single-cell RNAseq levels. However, transcriptomic data and actual protein validation convey conflicting information regarding the distribution of the biologically relevant protein receptor in whole tissues. To describe the organ-level architecture of receptor expression, related to the ability of ACE2 and TMPRSS2 to mediate infectivity, we performed a volumetric analysis of whole Syrian hamster lung lobes. Lung tissue of infected and control animals was stained using antibodies against ACE2 and TMPRSS2, combined with SARS-CoV-2 nucleoprotein staining. This was followed by light-sheet microscopy imaging to visualize their expression and related infection patterns. The data demonstrate that infection is restricted to sites containing both ACE2 and TMPRSS2, the latter is expressed in the primary and secondary bronchi whereas ACE2 is predominantly observed in the bronchioles and alveoli. Conversely, infection completely overlaps where ACE2 and TMPRSS2 co-localize in the tertiary bronchi, bronchioles, and alveoli.

## Author summary

The ongoing COVID19 pandemic necessitates additional tools to study SARS-CoV infection dynamics. This increases our understanding of where these viruses cause infections in the lung, and how essential different receptor molecules are. Here we applied tissue optical clearing and 3D immunofluorescence of whole organs from our best animal model, the Syrian hamster. We describe the organ-level architecture of receptor expression profiles, to visualize how their concerted action mediates SARS-CoV-2 infection patterns.

## Introduction

SARS-CoV-2 has sparked a pandemic and additional means to understand the infection dynamics of this virus will facilitate counter-measures. SARS coronaviruses carry a single protruding envelope protein, called spike, that is essential for binding to and subsequent infection of host cells. The trimeric spike protein binds to angiotensin-converting enzyme 2 (ACE2), which functions as an entry receptor for SARS-CoV [1,2]. After binding and internalization, TMPRSS2 induces the spike protein into its fusogenic form allowing fusion of the viral and target membrane. Several other attachment factors and/or receptors have been reported [3–6], but it is generally accepted that ACE2 and TMPRSS2 are essential. To understand how a zoonotic coronavirus is so successful in the human population, complementary techniques are required to describe the organ-level architecture of ACE2 and TMPRSS2 expression profiles, to describe how their concerted action mediates infectivity.

ACE2 and TMPRSS2 are expressed in a wide variety of tissues and have been analyzed using different genetic techniques [7–9]. High expression of these proteins was observed in extrapulmonary tissues, whereas the virus mainly infects the respiratory tract [7,8,10]. Conflicting transcriptomic data for ACE2 and TMPRSS2 expression in various tissues and cell types have been reported [7,8,10–13]. Several studies found that the majority of ACE2 was expressed in alveolar type II cells whilst others detected the highest expression in nasal epithelial cells followed by lower airway tissues [7,8,11]. In several studies, no ACE2 expression was observed in the gastrointestinal tract [14], whilst others found the highest expression in this tissue [8,10]. Even studies assessing actual biochemical expression of ACE2 portray conflicting data [10,12,13]. TMPRSS2 expression is described in fewer studies. Transcriptomic analyses indicated the highest expression in alveolar type I and II cells, followed by lung bronchus and in the nasal cavity [8]. High protein expression has been observed in bronchial epithelium and alveolar type II cells [13].

Beyond transcript concentration other factors contribute to protein expression, hence a direct correlation between mRNA and protein abundance for the same location/cell type may not reflect the actual situation [15]. Importantly, a deep proteome and transcriptome abundance atlas of 29 healthy human tissues assessing the expression and quantities of 13,640 human proteins confirmed that a majority of proteins with high mRNA expression were hardly detected in the proteome. For ACE2 and TMPRSS2 transcriptomic and proteomic data can be extracted from this publicly available dataset [10]. Initial comparison of ACE2 transcripts and ACE2 protein abundance already conveys a disparity, with low expression being observed in the appendix, lung and rectum whilst actual protein detection portray high expression in these tissues relative to actin (ACTB). TMPRSS2 transcript presence and protein abundance appeared to have a higher correlation. To determine whether two variables (transcripts and proteins) vary together the correlation coefficient can be assessed (S1 Fig). The direction and magnitude of correlation of several non-SARS-CoV-2 related genes (EIF4A3, ACTB and SYK) were determined as a baseline for ACE2 and TMPRSS2 correlation. For EIF4A3 there appeared to be no correlation (rho = -0.03) between transcript concentration and protein abundance, whilst ACTB displayed occasional correlation (rho = 0.52) and SYK showed an almost perfect correlation (rho = 0.92). ACE2 transcript and protein abundance correlation appeared to be occasional (rho = 0.45), whilst for TMPRSS2 the correlation was almost perfect (rho = 0.98). Although TMPRSS2 transcript presence and protein abundance appear to correlate better, a clear drawback of these genetic studies is that actual biochemical expression is not determined and mass-spectrometry-based assays do not retain spatial information.

High-resolution mapping of three-dimensional (3D) structures in intact tissues is indispensable in many biological studies, yet rarely employed to study viral receptors in their host organs concerning viral infection [16]. The conventional method of histological sectioning followed by the imaging of individual sections is commonly used and valuable, but does not provide spatial information, hampering our advancement of understanding viral infection spatially. Recent developments in whole organ clearing, imaging, and analysis of these large datasets do now allow for the characterization of whole organs [17]. Different animal models have been employed to recapitulate SARS-CoV-2 infection in humans, which are indispensable to develop vaccines and anti-viral therapeutics [18]. Several reviews succinctly compare the advantages and disadvantages of different animal models [19,20]. The Syrian hamster is widely accepted as the small animal model of choice [21–23]. Therefore an increased understanding of receptor expression in this model is of importance to study SARS-CoV-2 infection patterns, especially, since the new Omicron variant predominantly infects the upper respiratory tract and infection in the lung was absent [24–27].

Here, we examined receptor distribution in Syrian hamster lungs and correlated this distribution pattern of ACE2 and TMPRSS2 with the location of infection. Whole lung lobes of SARS-CoV-2 infected Syrian hamsters and control animals were stained using a variety of antibodies for ACE2, TMPRSS2, and the viral nucleoprotein to detect the functional receptor, cellular protease, and location of infection. We observed for ACE2 and TMPRSS2 a variation in protein expression levels within different regions of the lung lobes, with also a variation in protein expression between lung lobes from different Syrian hamsters. The results demonstrate that ACE2 and TMPRSS2 are unequally distributed in the Syrian hamster lung and that mainly overlapping regions were infected by SARS-CoV-2.

## Results

### Analysis of SARS-CoV-2 binding and receptors on lung tissue slides of Syrian hamsters lungs

Previously, we examined receptor binding of SARS-CoV trimeric receptor-binding domain (RBD) proteins to detect ACE2 in serial tissue slices of Syrian hamster lungs [28]. Here, we extended these studies by detecting TMPRSS2 to determine whether there are overlapping regions expressing both ACE2 and TMPRRS proteins and if potentially SARS-CoV-2 infection would occur in these sites of the lung. SARS-CoV-2 infection was observed only in the bronchioles and alveoli of infected Syrian hamster tissue slides (S2A Fig). Substantial ACE2 expression was detected in the alveoli and bronchioles of mock and 4-days post-infection (dpi) Syrian hamster lungs (S2B Fig). Next, we characterized TMPRSS2 expression; bronchiolar and alveolar staining was observed in non-infected Syrian hamster lungs, for infected Syrian hamster lungs staining was predominantly visualized in the bronchioles with minor alveolar staining (S2C Fig). For infected Syrian hamster lungs, overall reduced signal intensity was observed, in line with previous reports that demonstrated a possible downregulation of receptors and cellular proteases [28,29]. Additional staining was performed using an anti-keratin antibody, as keratin 8 (K8) and keratin 18 (K18) are expressed in human alveolar and bronchial epithelial cells [30,31]. Here this antibody was used to assess bronchiolar and alveolar staining of TMPRSS2, ACE2, and NP (S2D Fig). In non-infected tissue slides, K8/K18 was present in the alveoli and bronchioles (S2D Fig). In infected Syrian hamsters a distinct reduction of K8/K18 was observed, probably due to pulmonary damage. A secondary only antibody control was included with no apparent background fluorescence being detected (S2E Fig). Thus, we can detect SARS-CoV-2 infection/replication related to ACE2 and TMPRSS2 expression in different parts of the bronchiolar tree and alveolar sacs (S2F Fig). However, the use of tissue slides does not provide spatial information and prompted us to explore volumetric reconstructions instead.

### Syrian hamster lungs abundantly express ACE2 and TMPRSS2 in a distinct spatial manner

**Fig 1:**
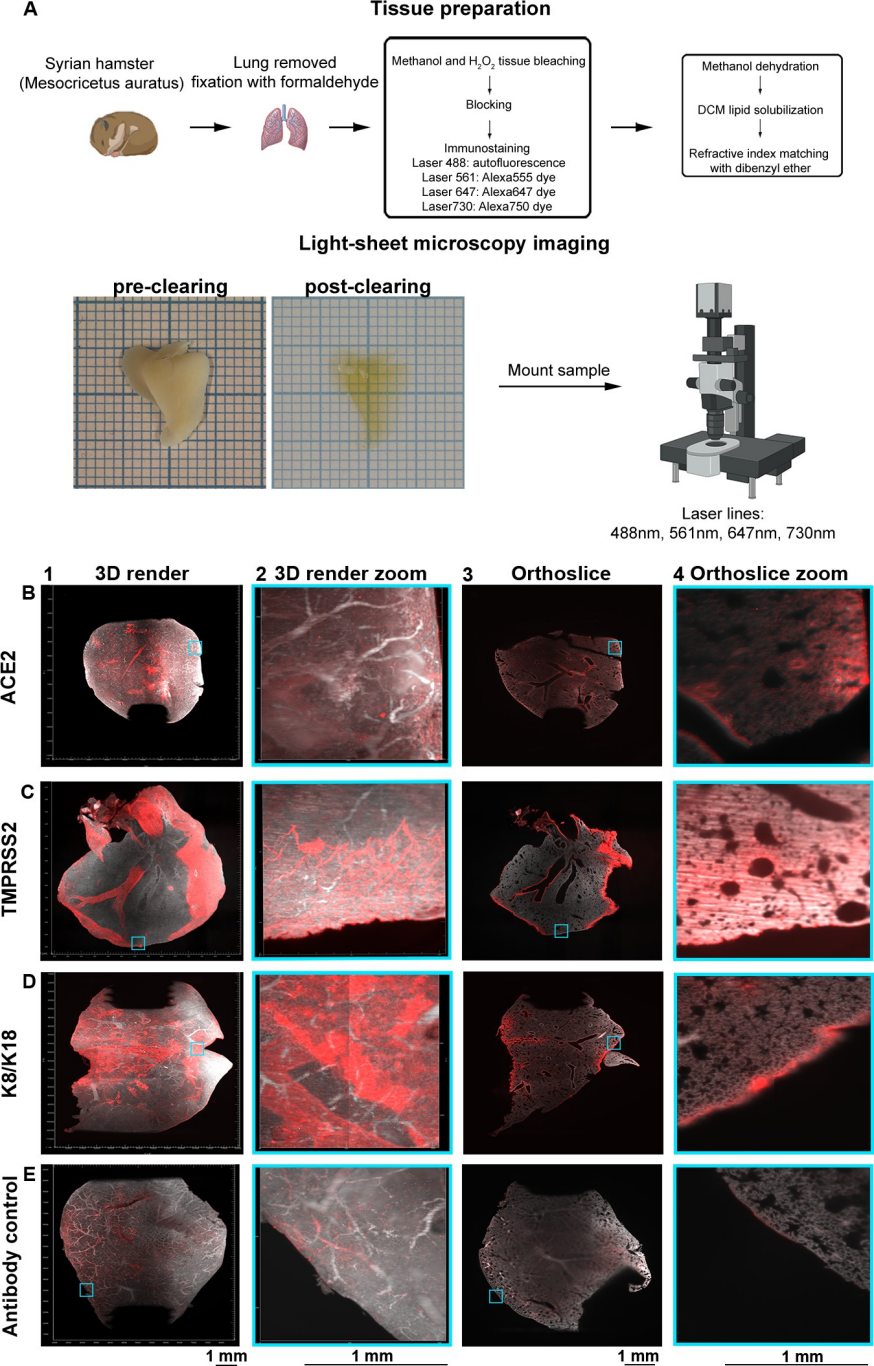
Volumetric analyzes of ACE2 and TMPRSS2 in non-infected Syrian hamster lung lobes delineate expression patterns. (A) Lung lobes from Syrian hamsters are extracted and fixated in 10% formalin. Tissue preparation is performed with bleaching, followed by blocking, immunostaining, dehydration, lipid solubilization, and refractive index matching. Refractive index matching for increased light penetration (pre-clearing vs. post-clearing on 1 mm paper), after which imaging is performed and data is analyzed. Orthogonal slices are generated with ImageJ and 3D renders with Imaris. Autofluorescence in grey (488 channel) and staining in red (647 channel). (B) ACE2 is distributed over the lung with nonuniform signals in the tertiary bronchi, bronchioles, and alveoli. (C) Significant TMPRSS2 expression in primary, secondary, and tertiary bronchi with minor alveolar presence. (D) Intense K8/K18 staining in the primary and secondary bronchi, with occasional signal in tertiary bronchi, bronchioles, and alveoli. (E) Autofluorescence in the 647 channel and minor staining of the outer regions of the lung for the secondary antibody control, the pattern does not overlap with previous stains.

Volumetric analysis was performed on Syrian hamster lung lobes to assess the spatial expression of ACE2 and TMPRSS2. The workflow of volumetric imaging and data analysis is described in Fig 1A. Syrian hamsters possess five lung lobes, for each staining we performed we used an individual lung lobe. ACE2 receptor expression was detected, with varying signal intensity, in tertiary bronchi, bronchioles, and alveoli (Fig 1B1–1B4). Hamster lung lobes incubated with anti-TMPRSS2 antibodies displayed intense staining in the secondary and tertiary bronchi with minor staining in the alveoli and substantial staining of the outer regions of the lung (Fig 1C1–1C4). In Fig 1D1 and 1D3, high signal intensity in the primary and secondary bronchus was observed when the anti-K8/K18 antibody was used, with occasional staining in several tertiary bronchi (Fig 1D1–1D4). Regions with alveolar staining were identified in the 3D render and orthoslice (Fig 1D2 and 1D4). Due to the extended antibody incubation periods using whole tissues, we employed a mixture of secondary antibodies only, in which a very minor background on the outside of tissues was observed, indicating a lack of a-specific binding while tissue penetration occured using primary antibodies (Fig 1E1–1E4). The detected fluorescent signals are presented without the autofluorescence channel in S3 Fig. Conclusively, we observed different spatial expression patterns of ACE2 and TMPRSS2, which encouraged us to expand our approach by simultaneously analyzing lung lobes for ACE2 and TMPRSS2.

### ACE2 and TMPRSS2 are unequally distributed in Syrian hamster lung lobes

**Fig 2:**
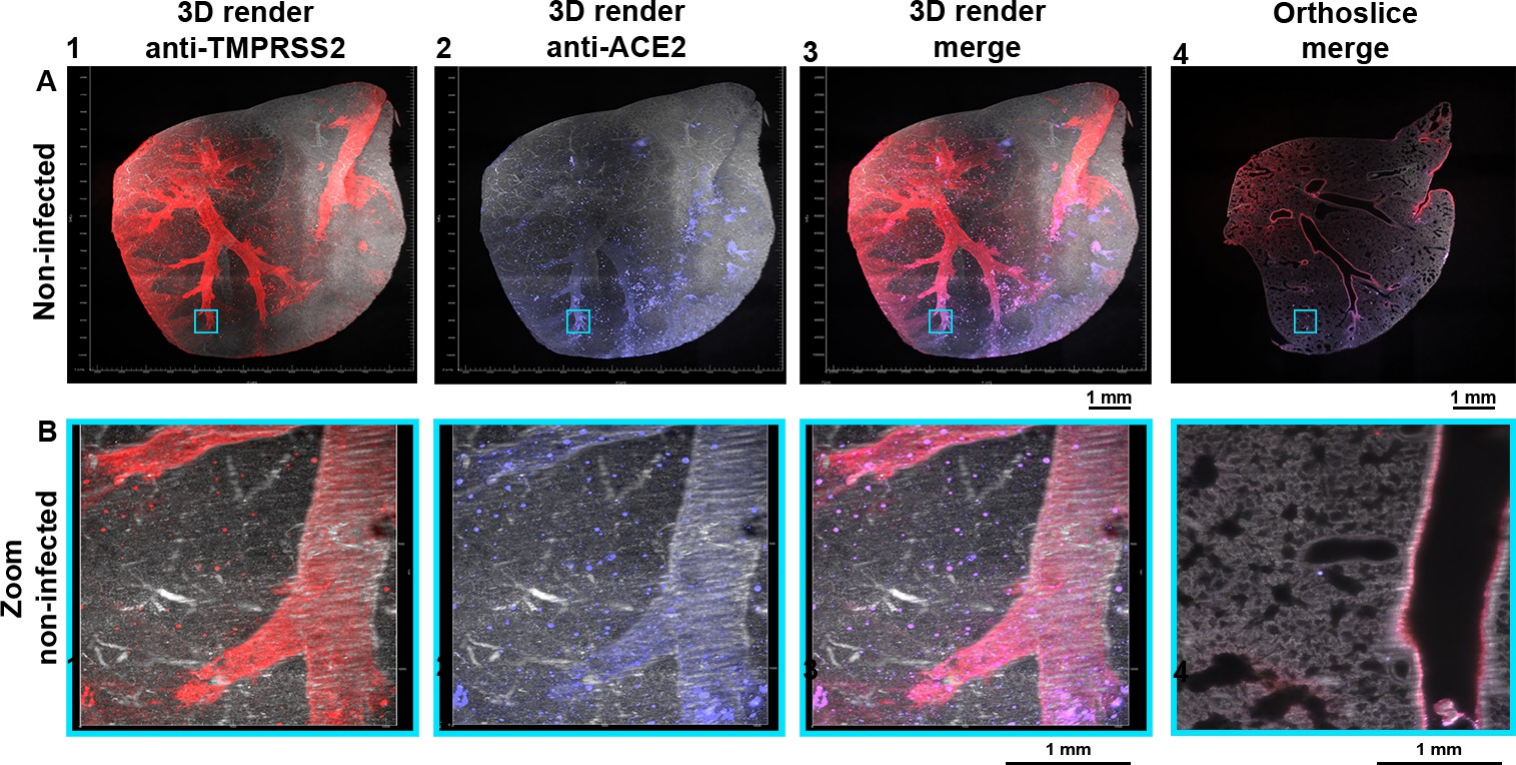
ACE2 and TMPRSS2 partially overlap. Autofluorescence in grey (488 channel), co-staining in red (647 channel), and purple (730 channel), with overlapping regions in pink (colorblind-proof). **(A)** TMPRSS2 expression is seen in the primary/secondary bronchi and several tertiary bronchi, with signal spreading to the bronchioles. ACE2 is predominantly located in the tertiary bronchi with relatively lower signals in the primary and secondary bronchi. With consistent expression for TMPRSS2 and bottom-to-top expression for ACE2. Overlap in several regions can be observed. 0.63 zoom, voxel resolution X,Y,Z: 4.79 μm, 4.79 μm, 5 μm. **(B)** Minor alveolar TMPRSS2 expression and mostly present at the transitioning point of the bronchioles to the alveolar sacs, a similar pattern is also observed for ACE2 expression. The expression profiles of TMPRSS2 and ACE2 overlap in the bronchioles and alveoli. 6.3 zoom, voxel resolution X,Y,Z: 0.48 μm, 0.48 μm, 2 μm. MP4 files of 3D render and orthoslice merge are provided.

To assess where potential virus binding and membrane fusion can occur, co-localization of ACE2 and TMPRSS2 was assessed in Syrian hamster lung lobes. Substantial staining was observed using the anti-TMPRSS2 antibody in the secondary and several tertiary bronchi (Figs 2A1 and S4). Signal dispersion to the bronchioles and alveoli was also detected with a similar pattern as observed in Fig 1, with minor alveolar staining at the transitioning point from bronchioles to the alveolar ducts (Figs 2B1 and S4B). ACE2 expression in this double-stained non-infected Syrian hamster lung was predominantly observed in tertiary bronchi with minor staining detected in the primary/secondary bronchi (Figs 2A2 and S4A). During both single and double stainings, expression of ACE2 was mainly observed in the bronchioles and alveoli (Figs 1B2 and 1B4, 2B2, and S4B). Substantial overlap was observed for TMPRSS2 and ACE2 in the tertiary bronchi and bronchioles (Figs 2A3, 2A4, 2B3, 2B4, S4A, and S4B and S1 and S2 Videos). In several regions (Figs 2A3, 2A4 and S4) no apparent overlap was detected, such as the primary/secondary bronchi where TMPRSS2 expression could be seen, and conversely in several bronchioles/alveolar ducts with ACE2 expression. Thus, ACE2 and TMPRSS2 are differentially expressed in the Syrian hamster lung lobes with TMPRSS2 staining consistently throughout the lung while for ACE2 a bottom-to-top gradient was detected.

### SARS-CoV-2 infected cells in whole Syrian hamster lung lobes spatially resolved

**Fig 3:**
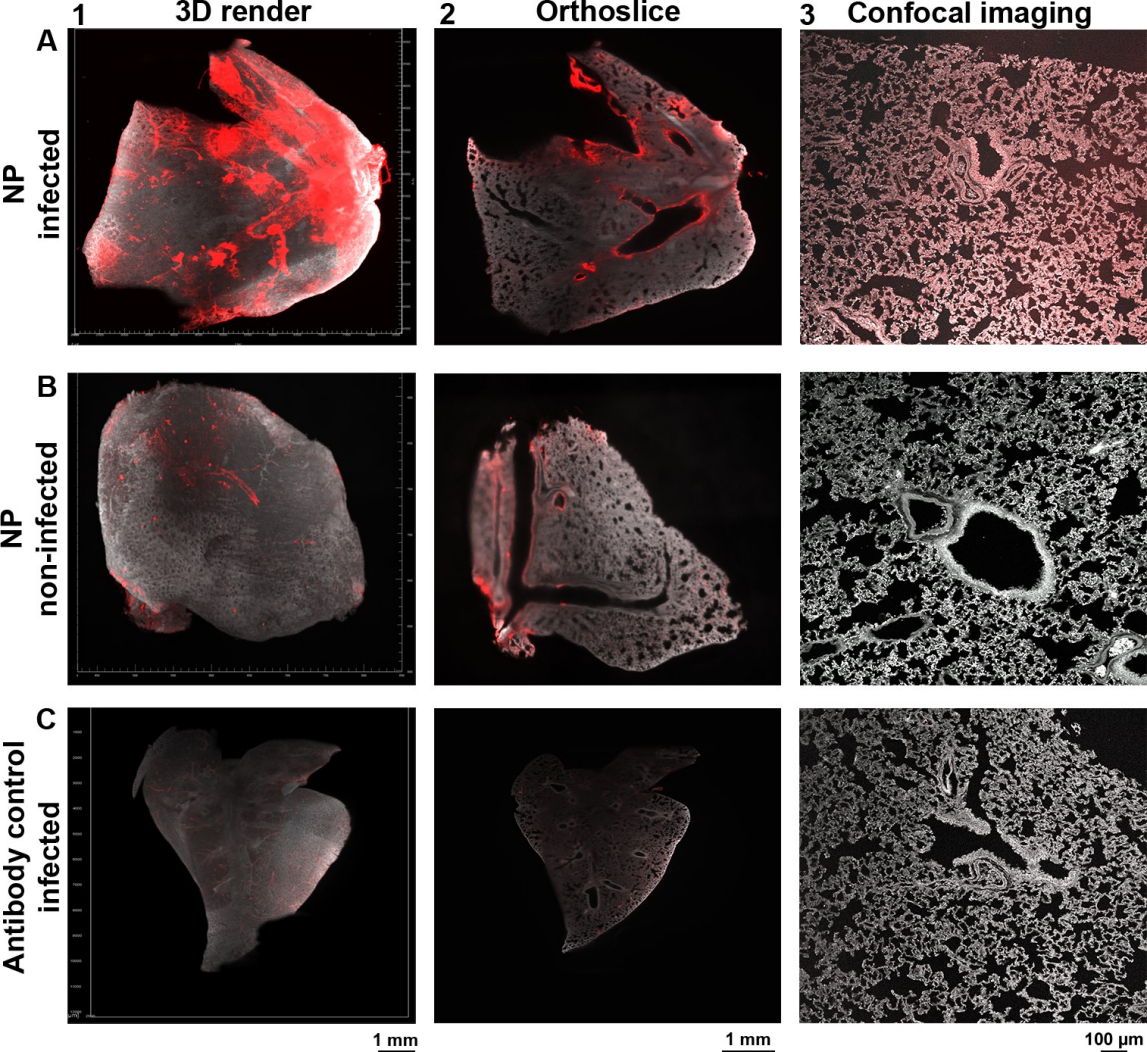
Detecting infected cells within a whole Syrian hamster lung lobe. Autofluorescence in grey (488 channel) and staining in red (647 channel). **(A)** Significant NP staining in the primary/secondary bronchi and bronchioles, with occasional tertiary bronchi and alveolar staining (light-sheet and confocal imaging data). **(B)** Non-specific binding in non-infected when using the anti-NP antibody in the bronchi and outer regions of the lung, although with a lower apparent signal intensity relative to infected lungs (light-sheet and confocal imaging data). **(C)** Minor autofluorescence and a-specific staining with secondary antibodies, the observed pattern does not overlap with previous stains (light-sheet and confocal imaging data).

To determine in which specific lung regions SARS-CoV-2 infection took place, we used hamster lung lobes isolated four days post-infection and stained these with anti-NP antibodies. High signal intensity was observed in the bronchi and bronchioles, however, several tertiary bronchi remained unstained (Figs 3A1 and 3A2, and S5A), and minor fluorescence signals in the alveoli (Fig 3A3). We also analyzed the anti-NP antibody on non-infected hamster lungs and observed some background at the major bronchi and on the outside regions of the lungs, albeit with an apparent lower fluorescence signal relative to the infected Syrian hamster lung lobes (Figs 3B1–3B3, S5A, and S5B). This was, however, not the case for other NP antibodies tested, for example, the 40143-R001 and MA1-7401 gave significant background staining through the bronchiolar tree (S6B and S6C Fig). Thus, the importance of adequate analysis of commercial antibodies cannot be overstated, as we have seen previously varying results with different anti-ACE2 antibodies [28]. The antibody control (secondary antibody only) for the infected hamster lung samples displayed minor background staining without any similarity to the NP stain (Figs 3C1–3C3 and S5C). Collectively, we can confidently detect infected regions within the whole lung lobes of infected Syrian hamsters.

### Location of infection is dictated to regions in which ACE2 and TMPRSS2 co-localize

**Fig 4:**
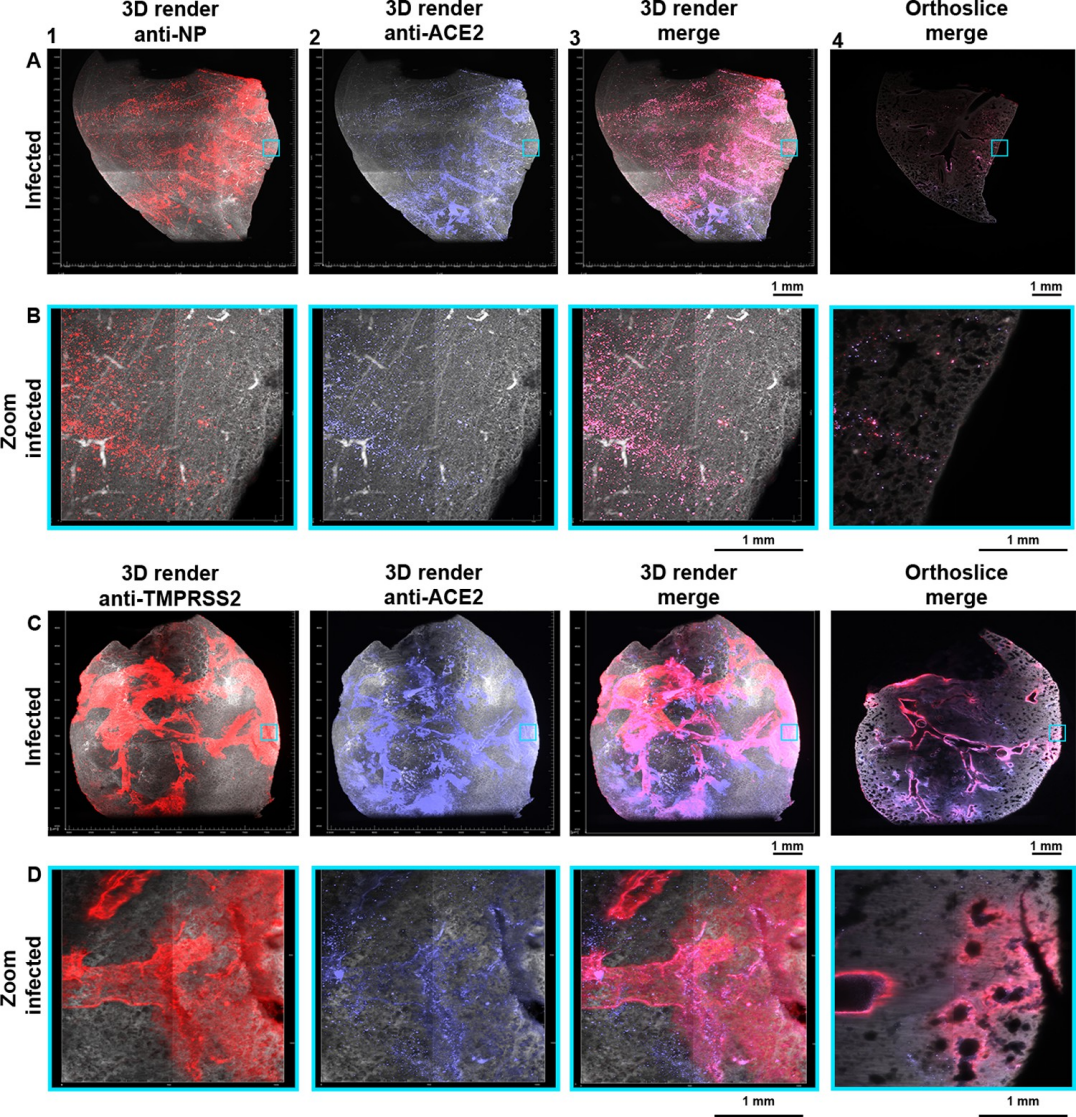
Visualizing tropism of SARS-CoV-2 infection with ACE2 and TMPRSS2 expression patterns at 4-dpi. Autofluorescence in grey (488 channel), co-staining in red (647 channel), and purple (730 channel), with overlapping regions in pink (colorblind-proof). **(A)** In several regions of the lung, NP staining is observed from the secondary bronchi extending to the tertiary bronchi, a top-to-bottom gradient. ACE2 is again a bottom-to-top gradient, with signals in the alveoli and various tertiary bronchi. There is a major overlap of NP with ACE2 in the tertiary bronchi, bronchioles, and alveoli (foci). In the lower portion of the lung, there is no apparent overlap of NP and ACE2. 0.63 zoom, voxel resolution X,Y,Z: 4.79 μm, 4.79 μm, 5 μm. **(B)** In the alveoli there is a significant overlap of NP and ACE2, the previously observed foci appear to be alveolar. Several NP-stained foci do not superimpose with ACE2 stained foci. 6.3 zoom, voxel resolution X,Y,Z: 0.48 μm, 0.48 μm, 2 μm. **(C)** The expression profile of TMPRSS2 in infected Syrian hamster lungs is highly similar to the expression in non-infected lungs. The fluorescence signal is spreading towards the bronchioles. Likewise, ACE2 expression is also highly similar to mock lungs, with protein staining in tertiary bronchi and bronchioles, 0.63 zoom, voxel resolution X,Y,Z: 4.79 μm, 4.79 μm, 5 μm. **(D)** At the transitioning point of bronchioles and alveoli TMPRSS2 and ACE2 superimpose, whilst in the alveoli only ACE2 expression is present. 6.3 zoom, voxel resolution X,Y,Z: 0.48 μm, 0.48 μm, 2 μm. MP4 files of 3D render and orthoslice merges are provided.

Being able to detect ACE2, TMPRSS2, and infected cells in a 3D format, we focused on examining overlapping regions of ACE2 and SARS-CoV-2 infection to assess if an infection is restricted to specific regions (Fig 4A and 4B). We observed NP staining in several regions of the secondary bronchi extending to tertiary bronchi and bronchioles (Figs 4A1 and S7A). The primary bronchi and the other secondary bronchi in the upper portion of the lung lobe appeared to be completely clear of infection. NP staining was observed in the alveoli, albeit at a relatively low signal intensity (Figs 4B1 and S7B), additionally over the entire lung a large number of foci were present (Figs 4B2 and S7B). ACE2 expression was again mainly detected in the tertiary bronchi in the lower parts of the lung lobe (Figs 4A2 and S7A). No significant signal was detected in the primary bronchi and the other secondary bronchi also appeared to express low levels of ACE2. ACE2 patterns were strikingly similar to the NP patterns, where the majority of the detected signal overlapped (Figs 4A3, 4B3, S7A, and S7B, and S3 and S4 Videos). NP staining appeared to overlap most strikingly with ACE2 staining in the tertiary bronchi, bronchioles, and alveoli (foci). However, ACE2 staining did not completely overlap with NP staining, in the lower regions of infected Syrian hamster lung lobes there appeared to be a lack of NP staining whilst clear ACE2 presence is observed. Thus, SARS-CoV-2 infection at four days post-infection, is restricted to the middle portion of the lung lobe, where receptors in the lower lung parts were not utilized. Next, we analyzed the spatial distribution of TMPRSS2 in relation to ACE2 in infected Syrian hamster lungs (Fig 4C and 4D). A similar expression profile was observed compared to the duplicate experiment in non-infected lungs (Fig 2). Substantial TMPRSS2 expression was detected in the primary, secondary and tertiary bronchi, with a spread to the bronchioles and alveoli, similar to previous single and double stains with alveolar staining at the transitioning point of the bronchioles and alveoli (Figs 4C1, 4D1, S7C, and S7D). ACE2 expression peaks enormously in the tertiary bronchi, bronchioles, and alveoli (Figs 4C2, 4D2, S7C, and S7D), with clearly lower signal in the primary and secondary bronchi (Figs 4C2 and S7C). The overlap of ACE2 and TMPRSS2 is predominantly present in the tertiary bronchi and the transitioning point of the bronchioles and alveoli (Figs 4C3, 4D3, S7C, and S7D, and S5 and S6 Videos). This overlap corresponded with the detection of NP staining, suggesting the necessity of both ACE2 and TMPRSS2 for infection.

**Fig 5:**
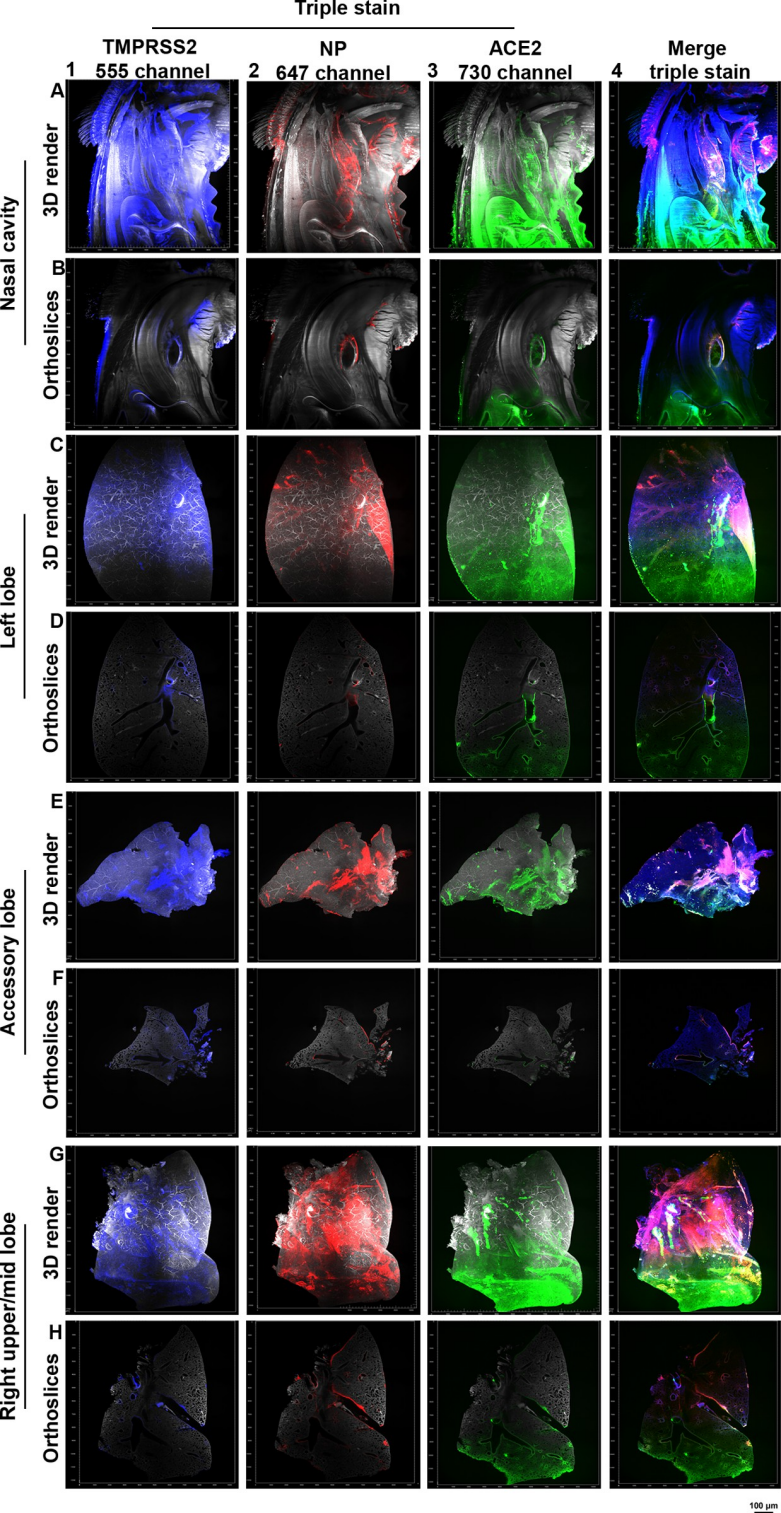
SARS-CoV-2 infection necessitates sufficient amounts of ACE2 and TMPRSS2 expression at 4-dpi. TMPRSS2 was imaged in the 561 channel, NP in the 647 channel, and ACE2 in the 730 channel. With fluorescence signal for the single channels shown in red (ACE2), green (NP), blue (TMPRSS2), and background in grey. (A/B) ACE2 and TMPRSS2 signals in the nasal cavity towards the nasopharynx overlaps with SARS-CoV-2 infection seen in both the 3D render and orthoslices. 0.63 zoom, voxel resolution X,Y,Z: 4.79 μm, 4.79 μm, 5 μm. (C/D) In the left lobe TMPRSS2 signal is seen in the larger branches and upper portions of the lung lobe spreading towards the alveoli. ACE2 signal is predominantly present in the alveoli, bronchioles, and tertiary branches lower in the lung lobe. SARS-CoV-2 infection is transposing in regions where both ACE2 and TMPRSS2 signal is present. (E/F) In the accessory lobe fluorescence signal of TMPRSS2, NP, and ACE2 is mostly visible in the larger branch, with no apparent signal in the alveoli. (G/H) The right upper/mid lobe contains TMPRSS2 signal in the larger branches with spread towards the alveoli in higher in the lung, whilst for ACE2 the signal is distributed mostly in the alveoli and lower portions of the lobe. Regions with SARS-CoV-2 infection, whereby high TMPRSS2 and low ACE2 are detected, also regions with low TMPRSS2 and high ACE2 signal.

After assessing ACE2 and NP overlap (Fig 4A and 4B), with also verifying the co-localization of ACE2 and TMPRSS2 (Figs 2, 4C, and 4D) we further characterized the expression patterns of both ACE2 and TMPRSS2 throughout the respiratory system, in combination with detection of SARS-CoV-2 infection (triple stain). In the previous experiments we did not use the 561 channel as light penetration through the cleared tissue is lower compared to the 647 nm lasers and up. In addition, data obtained in this 561 channel cannot be quantified since endogenous fluorescence skews the quantification. Albeit these potential drawbacks, we were able to detect significant TMPRSS2 and ACE2 signals in the nasal cavity of Syrian hamsters, which were present towards the opening of the nasopharynx, which also corresponds with SARS-CoV-2 infection (Fig 5A and 5B and S7 and S8 Videos orange arrow). In the triple staining lung lobes, ACE2 was mainly present in the tertiary bronchi in the lower parts of the lung lobes and TMPRSS2 expression was mainly observed in the larger branches whilst spreading towards the bronchioles and alveoli (Fig 5C–5H and S9–S14 Videos). SARS-CoV-2 infection is visualized in regions where ACE2 and TMPRSS2 expression is present (Fig 5A–5H panel 4), confirming the necessity of both proteins for infection. Noteworthy, the triple staining data, in combination with previous single and double staining data, suggests that the virus does not necessarily require significant expression of both ACE2 and TMPRSS2 to mediate infection, as seen in the left, accessory, and right upper/mid lobe. In certain regions low ACE2 and high TMPRSS2 expression is present (yellow arrow in S9–S14 Videos), with other regions where high ACE2 and low TMPRSS2 expression is observed (red arrow in S9–S14 Videos). Indicating the necessity of sufficient, not copious, amounts of receptor and cellular protease co-localization for SARS-CoV-2 infection. In S8 Fig, the stains are displayed without the autofluorescence channel.

### Statistical significance of ACE2 and TMPRSS2 spatial distribution in (non-) infected Syrian hamster lung lobes

**Fig 6:**
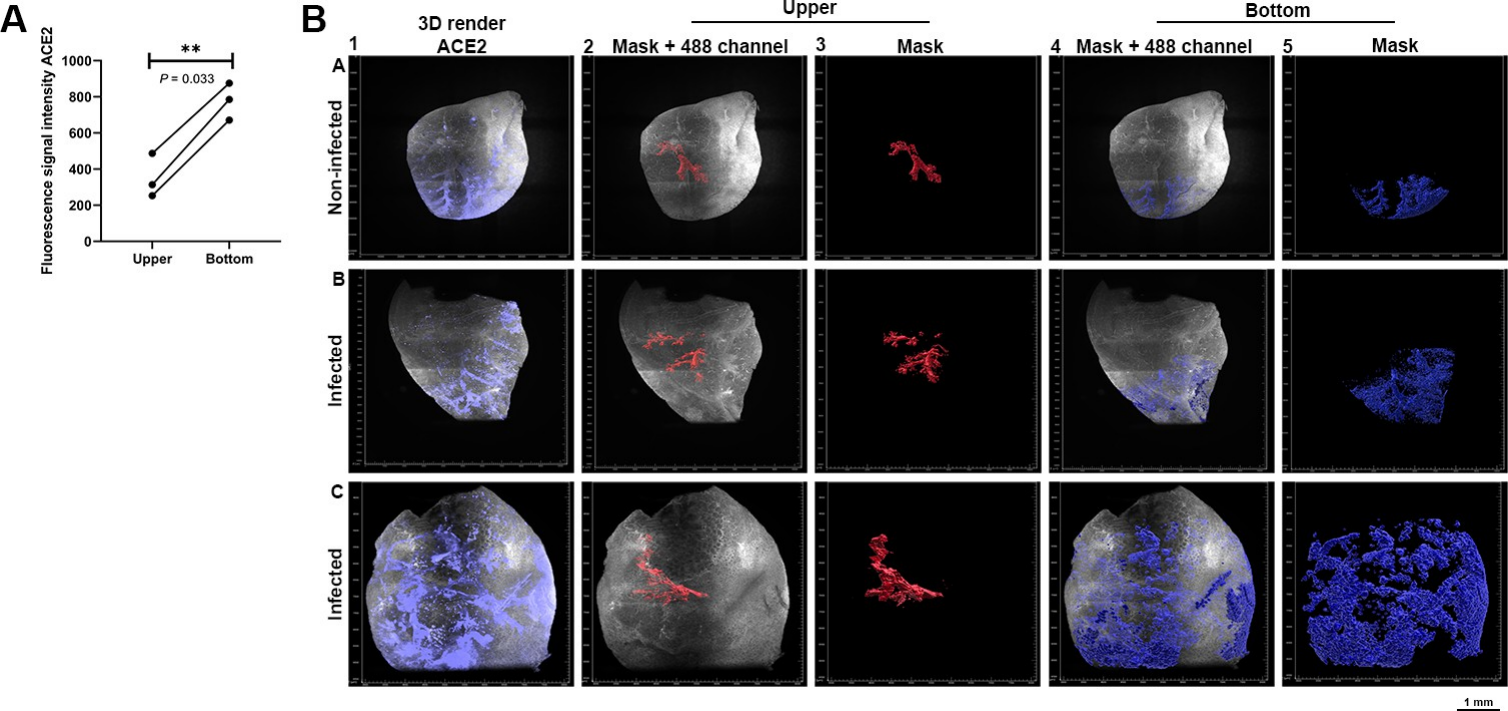
ACE2 is differentially expressed in (non-)infected Syrian hamster lung lobes. ACE2 signal shown in purple with autofluorescence (488 channel) in grey. The selected regions “upper” in red and “bottom” in blue. **(A)** Signals of “upper” and “bottom” were quantified, with significant differences in intensity between these regions (P = 0.033). **(B)** In panel A-C 1 3D render of ACE2 channel shown, hereafter a mask for the “upper” was generated, shown with and without the 488 channel (gray) in panel panel A-C 2–3. Masks for the “bottom” regions was also generated with and without the 488 channel in panel A-C 4–5. Selected regions were generated with the “Seed Points Diameter” tool in Imaris 9.7, after which all values were averaged for grouped analysis to assess the statistical significance.

We observed that TMPRSS2 and ACE2 are differentially expressed in Syrian hamster lung lobes, now we wanted to determine whether there is a statistical significance in the spatial distribution. TMPRSS2 expression is consistently present within the larger branches, bronchioles, and alveoli, whilst for ACE2 the expression is mainly observed in the bronchioles and alveoli. ACE2 expression gradients in infected Syrian hamster lungs appeared to be near identical to non-infected hamster lungs (Figs 2 and 4). We characterized this differential bottom-to-top expression in multiple lobes with various staining combinations, for which we also quantified the fluorescence intensity. The difference in fluorescence signal intensity is significant (*P* = 0.0033), whereby the signal is highest in the “bottom” (i.e. alveoli, bronchioles) and lowest in the “upper” region (i.e. primary/secondary branches) (Fig 6A). The signal intensities in the Syrian hamster lung lobes (Fig 6B panels A1-C1) were quantified by selecting “upper” (Fig 6B panels A2-C2 and A3-C3) and “bottom” regions and masking them for an average value (Fig 6B panels A4-C4 and A5-C5). TMPRSS2 signal intensity in the “upper” and “bottom” region was also assessed, although no tangible gradient in fluorescence intensity could be observed. Considering the significantly lower expression of ACE2 in the “upper” region (Fig 6) and the necessity of ACE2 and TMPRSS2 expression for infection (Figs 4A, 4B, and 5). We hypothesize that infection only occurs where ACE2 and TMPRSS2 are both present in sufficient density.

### The Syrian hamster vs. ferrets as an animal model: differential ACE2 and TMPRSS2 expression

**Fig 7:**
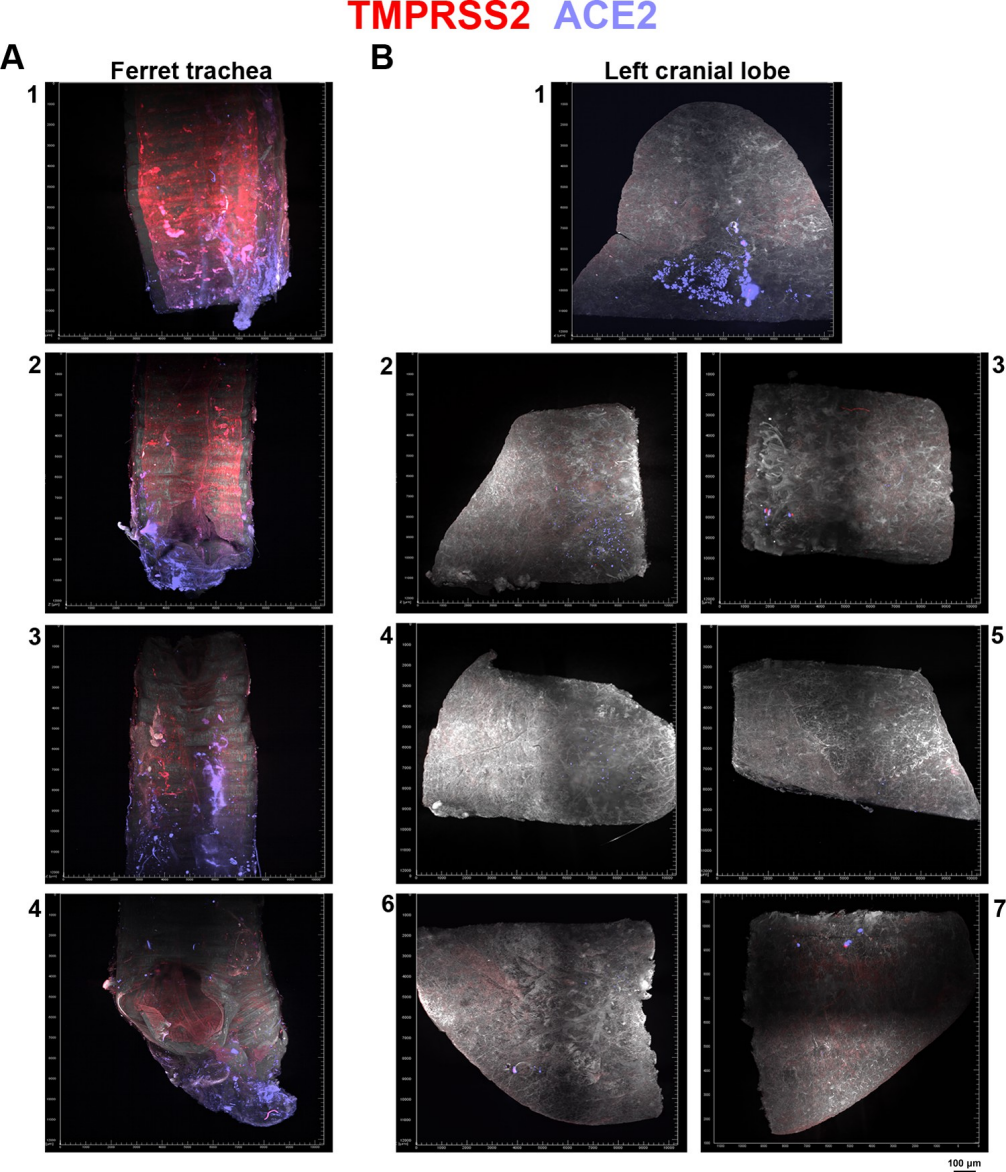
ACE2 and TMPRSS2 expression in the ferret animal model. TMPRSS2 fluorescence signal in red and ACE2 signal in purple. **(A)** In the ferret trachea, TMPRSS2 expression is observed with occasional ACE2 signal. Ferret trachea was cut into multiple pieces due to dimensional limitations of the microscope and after imaging and analysis the ferret trachea imaging data was placed in the order the ferret trachea was cut in, with panels 1–4 comprosing these cut trachea parts. Panel 1, part of the trachea that attaches to the ferret head with panel 4 being the part that is attached to the left cranial lobe. Panel 2 and 3 are parts of the trachea that are attached to the parts in panel 1 and 4. **(B)** In the left cranial lobe, minimal ACE2 expression is observed with no apparent TMPRSS2 signal. The left cranial lobe was cut into seven pieces, shown in panel 1–7. With panel 1 being the upper portion and panel 6/7 being the lower portion of the lobe, panel 2–5 are the pieces that connect panel 1 and 6/7 to form the entire cranial lung lobe.

The Syrian hamster is considered the most relevant animal model in studying SARS-CoV-2 infection since this model reflects infection and pathology in humans. In our study, we elaborated on the expression patterns of ACE2 and TMPRSS2 to assess the dependency of the virus for infection in a spatial context. In the literature, SARS-CoV-2 infection in ferrets is predominantly in the upper respiratory tract (URT). Indeed we observed significant ACE2 and TMPRSS2 levels in the ferret trachea, however, expression in the ferrets lung for both ACE2 and TMPRSS2 was minimal (Fig 7). Here we show the data of the ferret trachea and left cranial lung lobe that has been cut into multiple pieces due to microscope dimensional limitations. TMPRSS2 expression is relatively higher than ACE2 expression in the trachea where potential infection may occur. However, only minimal ACE2 expression is present in the left cranial lobe. Minimal ACE2 expression and lack of apparent TMPRSS2 signal in the left cranial lobe potentially explains the predominant URT SARS-CoV-2 infection in ferrets [32].

## Discussion

Transcriptomic data of ACE2 and TMPRSS2 expression has been informative, although a direct correlation between mRNA transcripts and protein abundance does not necessarily convey actual biochemical expression as described with the deep proteome analyses, also demonstrated by The Human Cell/Protein Atlas consortium [10] (S1 Fig). Beyond transcript concentration other factors contribute to protein expression, including translation rates influenced by gene codon composition, translation rate modulation through non-coding RNA (microRNA), Half-life modulation, protein synthesis delay, post-translational modifications, and protein transport, also residence time should be taken into account [15]. Therefore, imaging of ACE2, TMPRSS2, and infected cells in whole Syrian hamster lung lobes greatly contribute to our understanding of SARS-CoV-2 pathogenesis.

In this study, we demonstrate distinct spatial distribution, or gradient, of ACE2 and TMPRSS2 proteins in the lung lobes of Syrian hamsters, which is considered to be the most relevant small animal model for studying SARS-CoV-2 infection [19,21–23]. In most studies, whole Syrian hamster lungs have been analyzed to determine where SARS-CoV-2 infection occurs, and high viral loads have been detected in nasal and lung turbinate with similar lung histological changes as humans. Virus titers are normally determined from whole organs, and thus fail to give a detailed overview of infection dynamics in a spatial context. Furthermore, in these studies, whole lungs are subjected to tissue slicing, which may induce damage and result in a loss of spatial context when imaged in 2D. We now show the proteinaceous presence of the functional receptor ACE2, and the essential cellular protease TMPRSS2 [33–35], in concert with NP detection. We observed that in regions where ACE2 and TMPRSS2 overlap, actual infection occurs. Although other attachment factors such as sialic acids and heparan sulfate have been reported to be important for SARS-CoV-2 infection dynamics [3–6], NP always co-localizes with ACE2 in our study.

Light-sheet microscopy enables imaging of whole organs or large intact tissues in three dimensions. Previous implementations have focused on studying developmental biology or neuroscience [36], whilst this imaging technique also permits the investigation of viral dynamics in a native non-destructive 3D context [32]. Even though the spatial context is lost, tissue sectioning provides valuable information and functions as a complementary technique to light-sheet imaging to assess SARS-CoV-2 infection in relation to ACE2 and TMPRSS2 expression in bronchioles and alveoli. Using whole Syrian hamster heads and lung lobes with ferret trachea and lung lobes, we here provide a blueprint for analyzing infection patterns and receptor expression organization in a whole organ section. This approach will be valuable in identifying and testing new therapeutic agents for SARS-CoV-2. It will also provide opportunities to investigate extrapulmonary infection patterns and the approaches can be extended to other pathogens [37–39].

Finally, we envisage our approach can be applied to circulating variant viruses that are more infectious [40,41]. It can test a possible hypothesis that these viruses can infect the upper respiratory tract where the expression of ACE2 is rather scarce, resulting in increased transmission currently observed for the various variant of concern (VOC) [24]. Increased transmission of Omicron has been attributed to different ACE2 binding properties, differential dependency on cellular proteases [25], and indeed seems to infect upper respiratory tissues with higher efficiency compared to previous VOCs [26,27]. Lung infection in the Syrian hamster model is almost absent [42], with our imaging approach it is possible to characterize the infection pattern of Omicron in lung and URT. This model of adaptation to upper respiratory tract for increased transmission is extremely similar to the model for human influenza A viruses [43–45].

## Methods

### Ethics statement

Research was conducted in compliance with the Dutch legislation for the protection of animals used for scientific purposes (2014, implementing EU Directive 2010/63) and other relevant regulations. The licensed establishment where this research was conducted (Erasmus MC) has an approved OLAW Assurance # A5051-01. The research was conducted under a project license from the Dutch competent authority and the study protocol (#17–4312) was approved by the institutional Animal Welfare Body.

Animals were handled in a BSL3 biocontainment laboratory. Animals were housed in groups of 2 animals in filter top cages (T3, Techniplast), in Class III isolators allowing social interactions, under controlled conditions of humidity, temperature, and light (12-hour light/12-hour dark cycles). Food and water were available ad libitum. Animals were cared for and monitored (pre-and post-infection) by qualified personnel. The animals were sedated/anesthetized for all invasive procedures.

### Animal procedures SARS-CoV-2

Female Syrian golden hamsters (Mesocricetus auratus; 6-week-old hamsters from Janvier, France) were anesthetized by chamber induction (5 liters 100% O_2_/min and 3 to 5% isoflurane). Animals were inoculated with 10^5^ TCID50 of SARS-CoV-2 or PBS (mock controls) in a 100 μl volume via the intranasal route. Animals were monitored for general health status and behavior daily and were weighed regularly for the duration of the study (up to 22 days post-inoculation; d.p.i.). Animals were euthanized on day 4 after inoculation, and lung samples were removed and stored in 10% formalin for histopathology.

### Antibodies

The following commercial antibodies were purchased and utilized (S1 Table): anti-ACE2 antibody 2.5 μg/ml (Abcam, ab272690), anti-TMPRSS2 antibody 2.5 μg/ml (Santa Cruz, sc-515727), anti-NP 2.5 μg/ml (Sino-Biological, 40143-MM05), anti-NP 2.5 μg/ml (Sino-biological, 40143-R001), anti-NP 2.5 μg/ml (Thermofisher MA1-7401), anti-K8/K18 2.5 μg/ml (progen, 90001), donkey-anti-rabbit750 5.0 μg/ml (Abcam, ab175731), donkey-anti-GP647 2.5 μg/ml (Jackson immunoresearch, 706-605-148), goat anti-rabbit647 2.5 μg/ml (Thermofisher A-21245), goat-anti-mouse647 2.5 μg/ml (Thermofisher, A-21235), goat-anti-mouse555 2.5 μg/ml (A-21422), goat-anti-human647 2.5 μg/ml (Thermofisher, A-21445), goat-anti-rabbit488 10 μg/ml (Thermofsher, A-21432), goat-anti-human555 2.5 μg/ml (Thermofisher, A-21433). COVA103-1 antibody (10 μg/ml) was used as an anti-NP stain which was obtained from a convalescent patient. Biotinylated recombinant SARS-CoV-2 N proteins were conjugated with a streptavidin fluorophore resulting in fluorescent labeled-probes to stain N-specific B cells for flow cytometry sorting to obtain N-specific monoclonal antibody. The recombinant proteins were conjugated in a 7:1 ratio to the streptavidin-conjugates AF647 (0.5 mg/ml, BioLegend) and BV421 (0.1 mg/ml BioLegend). PBMCs from a convalescent patient were stained for 30 min at 4°C with the conjugated proteins and cell surface markers, as previously described [46]. Live B cells that were double-positive for the SARS-CoV-2 N protein were single-cell sorted using index sorting into a 96-well plate containing lysis buffer, stored at -80°C for at least 1 h before performing the reverse transcriptase (RT)-PCR followed by PCR amplification of the V(D)J variable regions, as previously described [47]. The amplified variable V(D)J-region of the heavy and light chain of the antibody were cloned into correspondingly expression vectors containing the constant regions of the human IgG1 for the heavy or light chain and larger-scale expression of the monoclonal antibody was done using transient transfection of suspension HEK293F cells (Invitrogen, cat no. R79007), as previously described [46].

### Tissue staining

Sections of formalin-fixed, paraffin-embedded Syrian hamster lungs were obtained from the department of Viroscience, Erasmus University Medical Center, The Netherlands. Tissue sections were rehydrated in a series of alcohol from 100%, 96% to 70%, and lastly in distilled water. Tissue slides were boiled in citrate buffer pH 6.0 for 10 min at 900 kW in a microwave for antigen retrieval and washed in PBS-T three times. Endogenous peroxidase activity was blocked with 1% hydrogen peroxide for 30 min. Tissues were subsequently incubated with 3% BSA in PBS-T overnight at 4°C. The next day, primary antibodies were added to the tissues for 1 h at RT. With rigorous washing steps in between followed by secondary antibodies with either an Alexa488, Alexa555, or Alexa647 fluorescent probe.

### IDISCO

SARS-CoV-2 infected and non-infected Syrian hamster lungs were provided in 10% formalin, for longer storage hamster lungs were kept in PBS and 0.01% sodium azide. Dehydration of the lungs was performed by washing with PBS for 1.5 hours, followed by 50% methanol for 1.5 hours, 80% methanol for 1.5 hours, and as of last 100% methanol for 1.5 hours on a tilting laboratory shaker. Bleaching was performed overnight at 4°C in 90% methanol (100% v/v) and 10% H_2_O_2_ (30% v/v). Tissues were rehydrated with 100% methanol for 1 hour, followed by 100% methanol for 1 hour, 80% methanol, 50% methanol, and 1x PBS for 1 hour on a tilting shaker. Syrian hamster lungs were subsequently blocked for 24 hours at room temperature (22°C) in 1x PBS with 0.2% gelatin, 0.5% triton-x-100, and 0.01% sodium azide (PBSGT) on a tilting shaker. Hamster lungs were stained with primary/secondary antibodies possessing an alexa555, alexa647, or alexa750 dye. After blocking samples were incubated for 120 hours with the primary antibody in PBSGT with 0.1% saponin (S2149, Sigma-Aldrich) on a shaking incubator at 200 rpm, following 120 hours incubation, hamster lungs were washed with PBSGT six times for 1 hour on a tilting shaker. Post-PBSGT wash, samples stained with primary antibodies were incubated with secondary antibodies that possess alexa555, alexa647 or alexa750 dyes, the antibodies were diluted in PBSGT with 0.1% saponin and filtered with 0.45 μm filter for potential antibody aggregates. Hereafter samples were incubated for 120 hours at room temperature on a shaking incubator at 200 rpm. After staining washing was performed with PBSGT six times for 1 hour on a tilting shaker. Immunostained samples were subsequently treated with 50% methanol for 24 hours, 80% methanol for 24 hours, 100% methanol for 24 hours followed by 100% methanol for 24 hours on a tilting shaker for dehydration of the lungs. Hereafter lipid solubilization was performed with dichloromethane (Thermofisher, 402152) for 40 minutes after which refractive index matching and optical clearing were performed overnight with dibenzyl ether (108014, Sigma-Aldrich).

### Light-sheet microscopy

Light-sheet imaging was performed using the Ultramicroscope II (LaVision BioTec) equipped with an MVX-10 Zoom Body. The laser lines 488 nm (Coherent OBIS 488–50 LX Laser 50mW), 561 nm (Coherent OBIS 561–100 LS Laser (100mW), 647 nm (Coherent OBIS 647–120 LX Laser 120mW), and 730 nm (Coherent OBIS 730–30 LX Laser 30mW) were used with 70% laser power for 488 channel, 70% laser power for the 555 channel, 70% laser power for the 647 channel and 100% laser power for the 730 nm channel. Emission filters ET525/50 (488 channel), ET615/40m (561 channel), ET676/29 (647 channel) and 716/40 (730 channel) filter was used with a Neo 5.5 sCMOS detector (2560x2160 pixels, pixel size: 6.5 x 6.5 μm^2^). A corrected dipping cap (CDC) (1.26x – 12.6x) was utilized with an Olympus MVPLAPO 2x objective lens. Imaging was performed with 0.63 zoom and step size of 5 μm (voxel resolution X, Y, Z: 4.79 μm, 4.79 μm, 5 μm), also with 6.3 zoom and step size of 2 μm (voxel resolution X, Y, Z: 0.48 μm, 0.48 μm, 2 μm). The exposure time was set to 200 ms and illumination for the 0.63 zoom was bidirectional and 6.3 zoom unidirectional. The sheet numerical aperture was 0.033 with a sheet thickness of 7.09 μm and a sheet width of 40%. The imaging chamber was filled with dibenzyl ether and the microscope software is Inspector version 7.1. Obtained image stacks were analyzed with ImageJ v1.54f and Imaris 9.7. Stills of image slices (orthoslices) were generated in ImageJ/Imaris, whilst 3D renders were generated in Imaris. TIFF files generated by the light-sheet microscope were imported to ImageJ with “File -> Import -> Bio-formats”, the stack is viewed as “Hyperstack” color mode was set to “Composite” and “Display metadata” with “Display OME-XML metadata” was enabled to obtain voxel information. Brightness adjustments were performed for each channel with setting “Image -> Adjust -> Brightness/Contrast”. The 488 channel color was set to hex code #FFFFFF, the 647 channel was set to hex code #FF0000 and the 730 channel was set to hex code #8080FF. Snapshots of the Z-stack were saved as JPEGs and the orthoslice animations with “Save As -> Avi -> Compression JPEG and Frame Rate 30 FPS”. For the 3D renders obtained imaging data was imported into Imaris, display adjustments were made for each channel. Snapshots and animations were made with 3D renders (3D View) within Imaris. Volume mode was set to maximum intensity projection (MIP) and the rendering quality was set to highest. Frame settings were used for the outer bounding box of the 3D render, “Box”, “Tickmarks”, and “Axis labels” were enabled. The spacing of the tickmarks was set to μm 500 for the X, Y, and Z-axis. The 488 channel color was set to hex code #FFFFFF, the 647 channel was set to hex code #FF0000 and the 730 channel was set to hex code #8080FF. For the triple stains, the 488 channel color was set to hex code #FFFFFF, the 561 channel color was set to hex code #0000FF, the 647 channel was set to hex code #FF0000 and the 730 channel was set to #00FF00. The 3D animations were made with 1920 x 1080 resolution (16:9) and 360 frames, 360° horizontal turn, and exported using Adobe Media Encoder 2020 or Adobe Premiere Pro 2020.

### Quantification

For TMPRSS2 and ACE2 quantification surfaces and masks were generated in Imaris with the “Surfaces Creation Wizard” (according to the reference manual). In this surface/mask regions of interest were selected, i.e. the branches and alveoli in the hamster lung. Regions of interest were selected in the surface tool with the corresponding source channel, whereby the 647 channel with TMPRSS2 signal was used for TMPRSS2 and ACE2 double-stained samples and 730 source channel for ACE2 and anti-NP double-stained samples. For the TMPRSS2 single stained sample, the 647 channel was used as the source channel. Thresholding (Absolute Intensity) was performed arbitrarily to obtain complete coverage of the alveoli and branches. The automatically provided value for “Sphere Diameter” was used. For the separation of two or more objects that are identified as one, the “Split touching Objects (Region Growing)” setting was utilized. The “Seed Points Diameter” value was set to 35 μm. The generated surfaces were subsequently used to mask the TMPRSS2 and ACE2 channels. The surfaces are split into multiple smaller regions within the surface, due to the setting “Seed Points Diameter”, hereafter all statistical values from these multiple smaller regions can be obtained and exported to GraphPad Prism 9 after which a grouped analysis was performed for statistical significance.

## Supporting information

S1 Fig Transcriptomic and proteomic correlation analysis of (non-) SARS-CoV-2 related genes. Spearman correlation coefficient (rho) calculated (each point represents a different tissue, e.g. lung or rectum) to assess whether the concentration of transcripts (FPKM) and protein abundance (IBAQ) vary together. P value provided, small p value rejects that correlation is due to random sampling. Large p value gives no compelling evidence that correlation is actually real and not due to chance. Intensity-based absolute quantification (IBAQ). Fragments Per Kilobase Million (FPKM)) **(A)** EIF4A3 gene has no correlation (rho = -0.03) with p = 0.88. **(B)** Occasional correlation for ACTB (rho = 0.52) with p = 0.0068. **(C)** Almost perfect correlation for SYK (rho = 0.92) with p < 0.0001. **(D)** ACE2 expression occasionally correlates (rho 0.45) with p = 0.0143. **(E)** For TMPRSS2 almost perfect correlation (rho = 0.98) with P < 0.0001
(TIF)

S2 Fig Expression of coronavirus receptors and co-factors on tissue sections of Syrian hamster lung. **(A)** No SARS-CoV-2 infection present in non-infected Syrian hamster lung (1–3), SARS-CoV-2 infection detected in the bronchioles and alveoli of infected Syrian hamster lungs (4–6). **(B)** ACE2 expression observed in Syrian hamster lungs. **(C)** TMPRSS2 expression is characterized, with reduced TMPRSS2 stain in the alveoli of infected Syrian hamsters (4–6). **(D)** K8/K18 stains with predominant bronchiolar staining. **(E)** With the secondary antibodies only, no staining is observed. **(F)** Overlap of ACE2 and TMPRSS2 is assessed in non-infected Syrian hamster lungs (1–3), with overlap of NP, ACE2 and TMPRSS2 in infected Syrian hamster lungs (4–6). **Red** arrow indicates no overlap while **blue** arrow indicates overlap.
(TIF)

S3 Fig 647 channel only supplementary data of non-infected single stains, related to Fig 1 staining in red (647 channel). **(A)** ACE2 distribution over Syrian hamster lung lobe. **(B)** TMPRSS2 signal present in larger branches. **(C)** K8/K18 staining in the primary, secondar and in tertiary bronchi, with bronchiolar and alveolar staining. **(D)** Autofluorescence in the antibody control.
(TIF)

S4 Fig Supplementary analyses of ACE2 and TMPRSS2 in a non-infected Syrian hamster lung, related to Fig 2. **(A)** TMPRSS2 expression in primary, secondary and tertiarty bronchi. Signal spreading to bronchioles. ACE2 staining in tertiary bronchi and bronchioles. In the tertiary bronchi overlap visualised of the receptor and co-factor 0.63 zoom, voxel resolution X,Y,Z: 4.79 μm, 4.79 μm, 5 μm. **(B)** Bronchiolar and alveolar TMPRSS2 and ACE2 expression visualized and superimposed. 6.3 zoom, voxel resolution X,Y,Z: 0.48 μm, 0.48 μm, 2 μm. **(Row 1)** 3D render 647 channel (Red hot false color). **(Row 2)** 3D render 730 channel (Fire false color). **(Row 3)** Merge 3D render 647 channel (Red hot false color) and 730 channel (Fire false color). **(Row 4)** Orthoslice merge 488 and 647 channel. **(Row 5)** Orthoslice 647 channel (Red hot false color). **(Row 6)** Orthoslice merge 488 and 730 channel. Row 7) orthoslice 730 channel (Fire false color). **(Row 8)** Merge orthoslice 647 channel (Red hot false color) and 730 channel (Fire false color). **(Row 9)** Orthoslice 488 channel (autofluorescence).
(TIF)

S5 Fig 488 and 647 channel only supplementary data of (non-)infected single stains, related to Fig 3. Light-sheet staining in red (647 channel) and confocal staining in red (488 channel). **(A)** NP staining in the primary/secondary bronchi. **(B)** Anti-NP antibody displays hardly non-specific binding in non-infected Syrian hamster lung lobes. **(C)** Autofluorescence in the antibody control.
(TIF)

S6 Fig Supplementary analysis of NP antibodies, related to Fig 4. Autofluorescence in grey (488 channel) and staining in red (647 channel). **(A)** Antibody 40143-MM05 on non-infected Syrian hamster lungs shows non-specific interaction towards the outer regions of the lung lobe. **(B)** Intense staining with antibody 40143-R001 in a Syrian hamster lung lobe, severe non-specific binding is observed. **(C)**Non-specific interaction of antibody MA1-7401 in a lung lobe whereby similar bronchiolar structures as infected hamster lungs are stained.
(TIF)

S7 Fig Supplementary analyses of NP, ACE2 and TMPRSS2 in a 4-dpi Syrian hamster lung, related to Fig 4. **(A)** Top-to-bottom NP stain, signal in the secondary bronchi extending towards the tertiary bronchi. A bottom-to-top gradient for ACE2 stain, with signal in the secondary and various tertiary bronchi. Overlap of NP and ACE2 in tertiary bronchi and bronchioles. 0.63 zoom, voxel resolution X,Y,Z: 4.79 μm, 4.79 μm, 5 μm. **(B)** NP and ACE2 foci overlayed, severe overlap is visualized in the alveoli. 6.3 zoom, voxel resolution X,Y,Z: 0.48 μm, 0.48 μm, 2 μm. **(C)** TMPRSS2 expression profile, predominant staining in the primary, secondary and tertiary bronchi with signal spreading towards the bronchioles. ACE2 and TMPRSS2 superimpose in the primarily in the tertiary bronchi and bronchioles 0.63 zoom, voxel resolution X,Y,Z: 4.79 μm, 4.79 μm, 5 μm. **(D)** Overlap of TMPRSS2 and ACE2 only in the bronchioles, whilst ACE2 expression is also present in the alveoli. 6.3 zoom, voxel resolution X,Y,Z: 0.48 μm, 0.48 μm, 2 μm**. (Row 1)** 3D render 647 channel (Red hot false color). **(Row 2)** 3D render 730 channel (Fire false color). **(Row 3)** Merge 3D render 647 channel (Red hot false color) and 730 channel (Fire false color). **(Row 4)** Orthoslice merge 488 and 647 channel. **(Row 5)** Orthoslice 647 channel (Red hot false color). **(Row 6)** Orthoslice merge 488 and 730 channel. **(Row 7)** Orthoslice 730 channel (Fire false color). **(Row 8)** Merge orthoslice 647 channel (Red hot false color) and 730 channel (Fire false color). **(Row 9)** Orthoslice 488 channel (autofluorescence).
(TIF)

S8 Fig SARS-CoV-2 infection releated to ACE2 and TMPRSS2 protein distribution at 4-dpi, related to Fig 5. TMPRSS2 imaged in the 555 channel, NP in the 647 channel and ACE2 in the 730 channel. Merges of all channels shown in red (ACE2), green (NP) and blue (TMPRSS2). **(A/B)** ACE2 and TMPRSS2 signal in the nasal cavity with SARS-CoV-2 infection overlap. 0.63 zoom, voxel resolution X,Y,Z: 4.79 μm, 4.79 μm, 5 μm. **(C/D)** In the left lobe TMPRSS2 signal is seen in the upper portions of the lung lobe. ACE2 signal mostly lower in the lung lobe. SARS-CoV-2 infection is transposing in regions where both ACE2 and TMPRSS2 signal is present. **(E/F)** In the accessory lobe fluorescence signal of TMPRSS2, NP and ACE2 overlap. **(G/H)** The right upper/mid lobe contains TMPRSS2, ACE2 and NP signal superimpose. Regions with SARS-CoV-2 infection either show high TMPRSS2 and low ACE2 signal or low TMPRSS2 and high ACE2.
(TIF)

S1 Table Syrian hamster lung lobes antibody staining combinations. Lung lobes from multiple Syrian hamsters have been stained with various primary and secondary antibodies. For co-staining experiments two primary and two secondary antibodies were utilized. For triple stain three primary and three secondary antibodies were used.
(XLSX)

S1 Video 3D render of ACE2 and TMPRSS2 spatial overlap in non-infected Syrian hamster lung lobes, related to Fig 2. ACE2 in purple and TMPRSS2 in red with overlap in pink.
(MP4)

S2 Video Orthoslice render of ACE2 and TMPRSS2 spatial overlap in non-infected Syrian hamster lung lobes, related to Fig 2. ACE2 in purple and TMPRSS2 in red with overlap in pink.
(MP4)

S3 Video 3D render of ACE2 and SARS-CoV-2 co-localization in infected Syrian hamster lung lobes, related to Fig 4. ACE2 in purple and SARS-CoV-2 infection in red with overlap in pink.
(MP4)

S4 Video Orthoslice render of ACE2 and SARS-CoV-2 co-localization in infected Syrian hamster lung lobes, related to Fig 4. ACE2 in purple and SARS-CoV-2 infection in red with overlap in pink.
(MP4)

S5 Video 3D render of ACE2 and TMPRSS2 spatial overlap in infected Syrian hamster lung lobes, related to Fig 4. ACE2 in purple and TMPRSS2 in red with overlap in pink.
(MP4)

S6 Video Orthoslice render of ACE2 and TMPRSS2 spatial overlap in infected Syrian hamster lung lobes, related to Fig 4. ACE2 in purple and TMPRSS2 in red with overlap in pink.
(MP4)

S7 Video 3D render of ACE2, TMPRSS2 and SARS-CoV-2 infection overlap in nasal cavity of infected Syrian hamster, related to Fig 5. In red ACE2, in green SARS-CoV-2 and in blue TMPRSS2 with overlapping regions in white.
(MP4)

S8 Video Orthoslice render of ACE2, TMPRSS2 and SARS-CoV-2 infection overlap in nasal cavity of infected Syrian hamster, related to Fig 5. In red ACE2, in green SARS-CoV-2 and in blue TMPRSS2 with overlapping regions in white.
(MP4)

S9 Video 3D render of ACE2, TMPRSS2 and SARS-CoV-2 infection overlap in left lobe of infected Syrian hamster, related to Fig 5. In red ACE2, in green SARS-CoV-2 and in blue TMPRSS2 with overlapping regions in white. Yellow arrow indicates regions with low ACE2 and high TMPRSS2 expression. Red arrow indicates regions with high ACE2 and low TMPRSS2 expression.
(MP4)

S10 Video Orthoslice render of ACE2, TMPRSS2 and SARS-CoV-2 infection overlap in left lobe of infected Syrian hamster, related to Fig 5. In red ACE2, in green SARS-CoV-2 and in blue TMPRSS2 with overlapping regions in white. Yellow arrow indicates regions with low ACE2 and high TMPRSS2 expression. Red arrow indicates regions with high ACE2 and low TMPRSS2 expression.
(MP4)

S11 Video 3D render of ACE2, TMPRSS2 and SARS-CoV-2 infection overlap in right/mid lobe of infected Syrian hamster, related to Fig 5. In red ACE2, in green SARS-CoV-2 and in blue TMPRSS2 with overlapping regions in white. Yellow arrow indicates regions with low ACE2 and high TMPRSS2 expression. Red arrow indicates regions with high ACE2 and low TMPRSS2 expression.
(MP4)

S12 Video Orthoslice render of ACE2, TMPRSS2 and SARS-CoV-2 infection overlap in right/mid lobe of infected Syrian hamster, related to Fig 5. In red ACE2, in green SARS-CoV-2 and in blue TMPRSS2 with overlapping regions in white. Yellow arrow indicates regions with low ACE2 and high TMPRSS2 expression. Red arrow indicates regions with high ACE2 and low TMPRSS2 expression.
(MP4)

S13 Video 3D render of ACE2, TMPRSS2 and SARS-CoV-2 infection overlap in accessory lobe of infected Syrian hamster, related to Fig 5. In red ACE2, in green SARS-CoV-2 and in blue TMPRSS2 with overlapping regions in white. Yellow arrow indicates regions with low ACE2 and high TMPRSS2 expression. Red arrow indicates regions with high ACE2 and low TMPRSS2 expression.
(MP4)

S14 Video Orthoslice render of ACE2, TMPRSS2 and SARS-CoV-2 infection overlap in accessory lobe of infected Syrian hamster, related to Fig 5. In red ACE2, in green SARS-CoV-2 and in blue TMPRSS2 with overlapping regions in white. Yellow arrow indicates regions with low ACE2 and high TMPRSS2 expression. Red arrow indicates regions with high ACE2 and low TMPRSS2 expression.
(MP4)
