# Supplementary figures and images for: Distinct spatial arrangements of ACE2 and TMPRSS2 expression in Syrian hamster lung lobes dictates SARS-CoV-2 infection patterns
Source: PLoS Pathog. 2022 Mar 7;18(3):e1010340. doi: 10.1371/journal.ppat.1010340 (PMC8930000; doi:10.1371/journal.ppat.1010340)

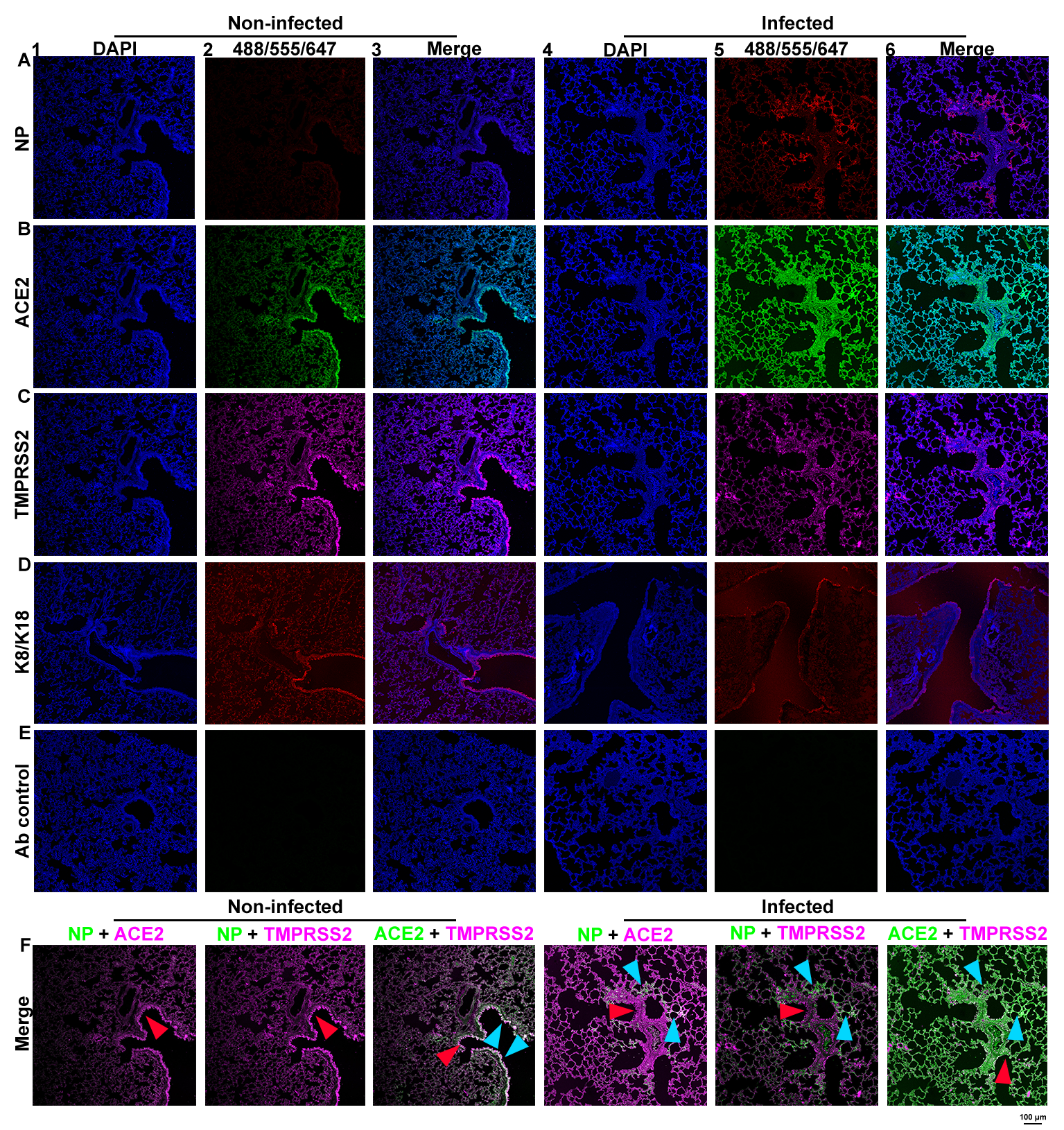

Supplement: S2 Fig — (A) No SARS-CoV-2 infection present in non-infected Syrian hamster lung (1–3), SARS-CoV-2 infection detected in the bronchioles and alveoli of infected Syrian hamster lungs (4–6). (B) ACE2 expression observed in Syrian hamster lungs. (C) TMPRSS2 expression is characterized, with reduced TMPRSS2 stain in the alveoli of infected Syrian hamsters (4–6). (D) K8/K18 stains with predominant bronchiolar staining. (E) With the secondary antibodies only, no staining is observed. (F) Overlap of ACE2 and TMPRSS2 is assessed in non-infected Syrian hamster lungs (1–3), with overlap of NP, ACE2 and TMPRSS2 in infected Syrian hamster lungs (4–6). Red arrow indicates no overlap while blue arrow indicates overlap. (TIF) [file ppat.1010340.s002.tif]

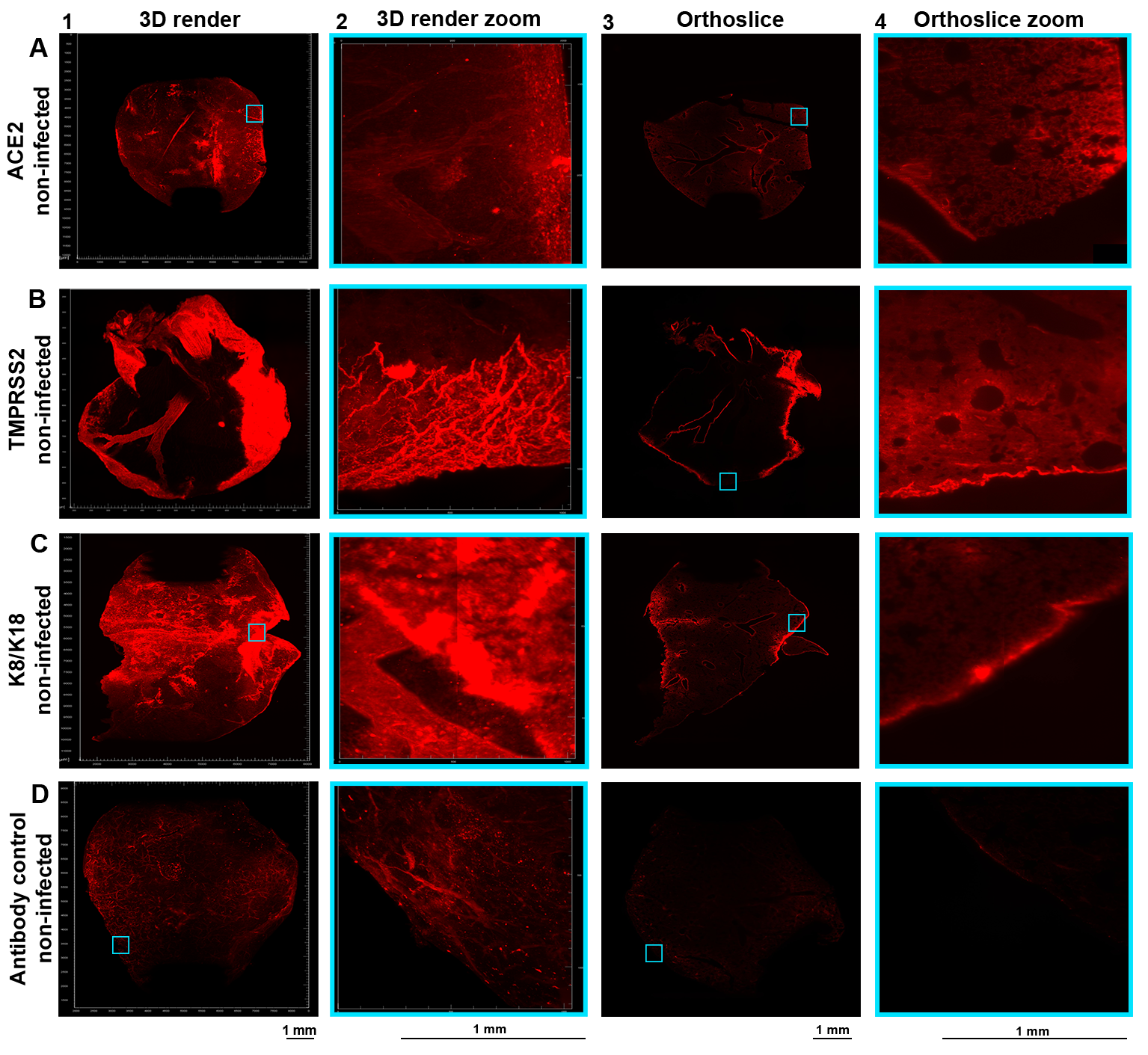

Supplement: S3 Fig — (A) ACE2 distribution over Syrian hamster lung lobe. (B) TMPRSS2 signal present in larger branches. (C) K8/K18 staining in the primary, secondar and in tertiary bronchi, with bronchiolar and alveolar staining. (D) Autofluorescence in the antibody control. (TIF) [file ppat.1010340.s003.tif]

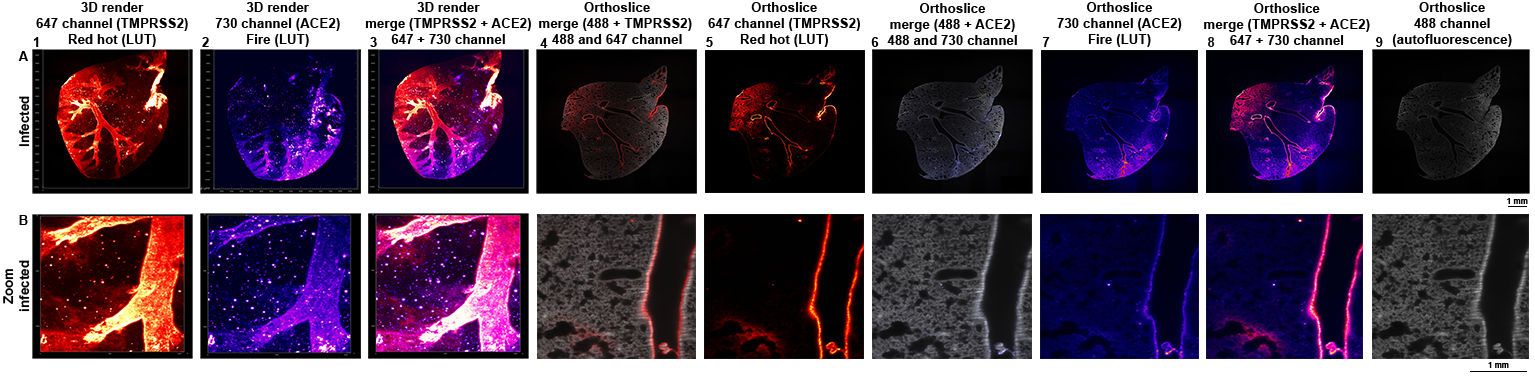

Supplement: S4 Fig — (A) TMPRSS2 expression in primary, secondary and tertiarty bronchi. Signal spreading to bronchioles. ACE2 staining in tertiary bronchi and bronchioles. In the tertiary bronchi overlap visualised of the receptor and co-factor 0.63 zoom, voxel resolution X,Y,Z: 4.79 μm, 4.79 μm, 5 μm. (B) Bronchiolar and alveolar TMPRSS2 and ACE2 expression visualized and superimposed. 6.3 zoom, voxel resolution X,Y,Z: 0.48 μm, 0.48 μm, 2 μm. (Row 1) 3D render 647 channel (Red hot false color). (Row 2) 3D render 730 channel (Fire false color). (Row 3) Merge 3D render 647 channel (Red hot false color) and 730 channel (Fire false color). (Row 4) Orthoslice merge 488 and 647 channel. (Row 5) Orthoslice 647 channel (Red hot false color). (Row 6) Orthoslice merge 488 and 730 channel. Row 7) orthoslice 730 channel (Fire false color). (Row 8) Merge orthoslice 647 channel (Red hot false color) and 730 channel (Fire false color). (Row 9) Orthoslice 488 channel (autofluorescence). (TIF) [file ppat.1010340.s004.tif]

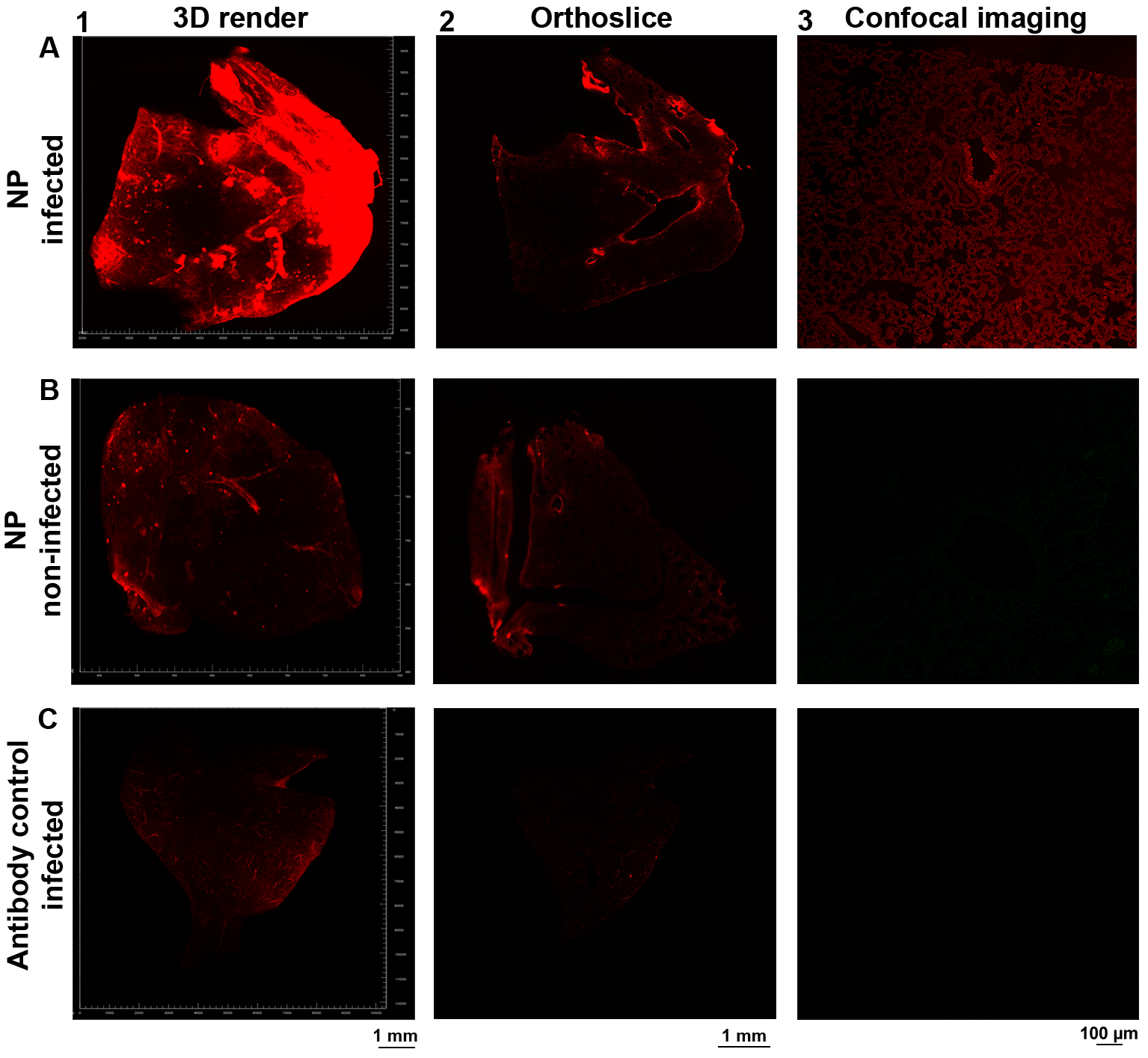

Supplement: S5 Fig — Light-sheet staining in red (647 channel) and confocal staining in red (488 channel). (A) NP staining in the primary/secondary bronchi. (B) Anti-NP antibody displays hardly non-specific binding in non-infected Syrian hamster lung lobes. (C) Autofluorescence in the antibody control. (TIF) [file ppat.1010340.s005.tif]

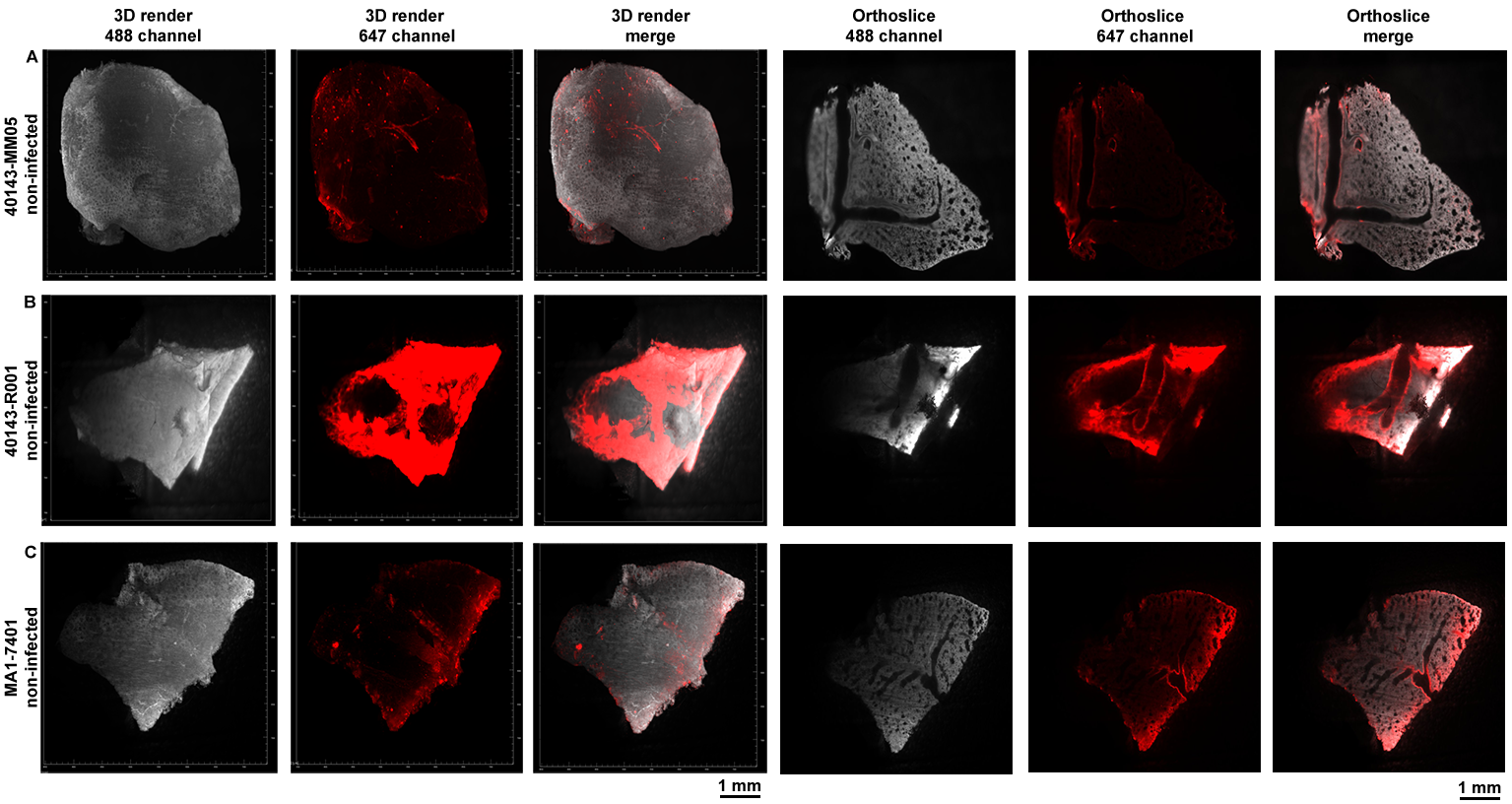

Supplement: S6 Fig — Autofluorescence in grey (488 channel) and staining in red (647 channel). (A) Antibody 40143-MM05 on non-infected Syrian hamster lungs shows non-specific interaction towards the outer regions of the lung lobe. (B) Intense staining with antibody 40143-R001 in a Syrian hamster lung lobe, severe non-specific binding is observed. (C)Non-specific interaction of antibody MA1-7401 in a lung lobe whereby similar bronchiolar structures as infected hamster lungs are stained. (TIF) [file ppat.1010340.s006.tif]

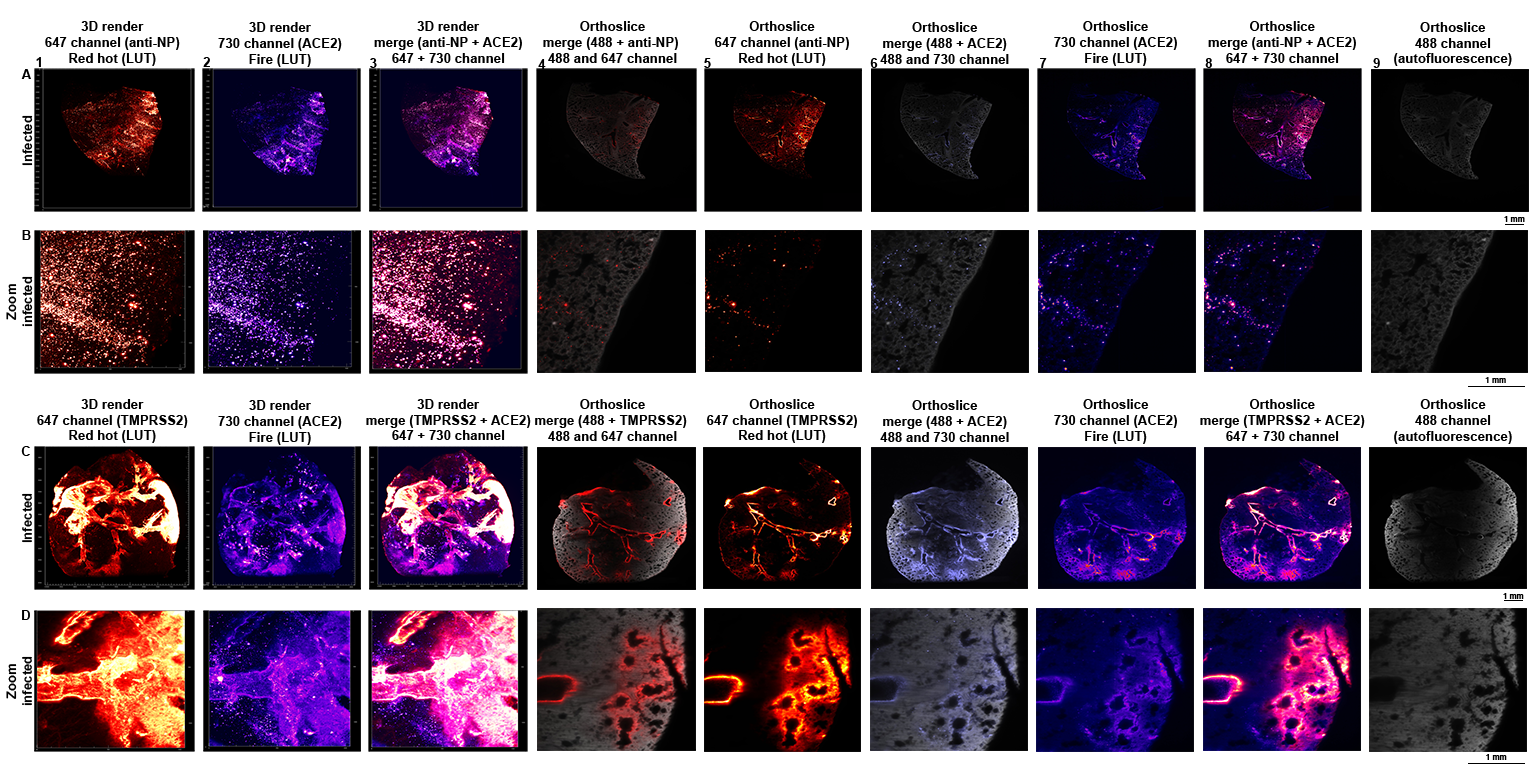

Supplement: S7 Fig — (A) Top-to-bottom NP stain, signal in the secondary bronchi extending towards the tertiary bronchi. A bottom-to-top gradient for ACE2 stain, with signal in the secondary and various tertiary bronchi. Overlap of NP and ACE2 in tertiary bronchi and bronchioles. 0.63 zoom, voxel resolution X,Y,Z: 4.79 μm, 4.79 μm, 5 μm. (B) NP and ACE2 foci overlayed, severe overlap is visualized in the alveoli. 6.3 zoom, voxel resolution X,Y,Z: 0.48 μm, 0.48 μm, 2 μm. (C) TMPRSS2 expression profile, predominant staining in the primary, secondary and tertiary bronchi with signal spreading towards the bronchioles. ACE2 and TMPRSS2 superimpose in the primarily in the tertiary bronchi and bronchioles 0.63 zoom, voxel resolution X,Y,Z: 4.79 μm, 4.79 μm, 5 μm. (D) Overlap of TMPRSS2 and ACE2 only in the bronchioles, whilst ACE2 expression is also present in the alveoli. 6.3 zoom, voxel resolution X,Y,Z: 0.48 μm, 0.48 μm, 2 μm. (Row 1) 3D render 647 channel (Red hot false color). (Row 2) 3D render 730 channel (Fire false color). (Row 3) Merge 3D render 647 channel (Red hot false color) and 730 channel (Fire false color). (Row 4) Orthoslice merge 488 and 647 channel. (Row 5) Orthoslice 647 channel (Red hot false color). (Row 6) Orthoslice merge 488 and 730 channel. (Row 7) Orthoslice 730 channel (Fire false color). (Row 8) Merge orthoslice 647 channel (Red hot false color) and 730 channel (Fire false color). (Row 9) Orthoslice 488 channel (autofluorescence). (TIF) [file ppat.1010340.s007.tif]

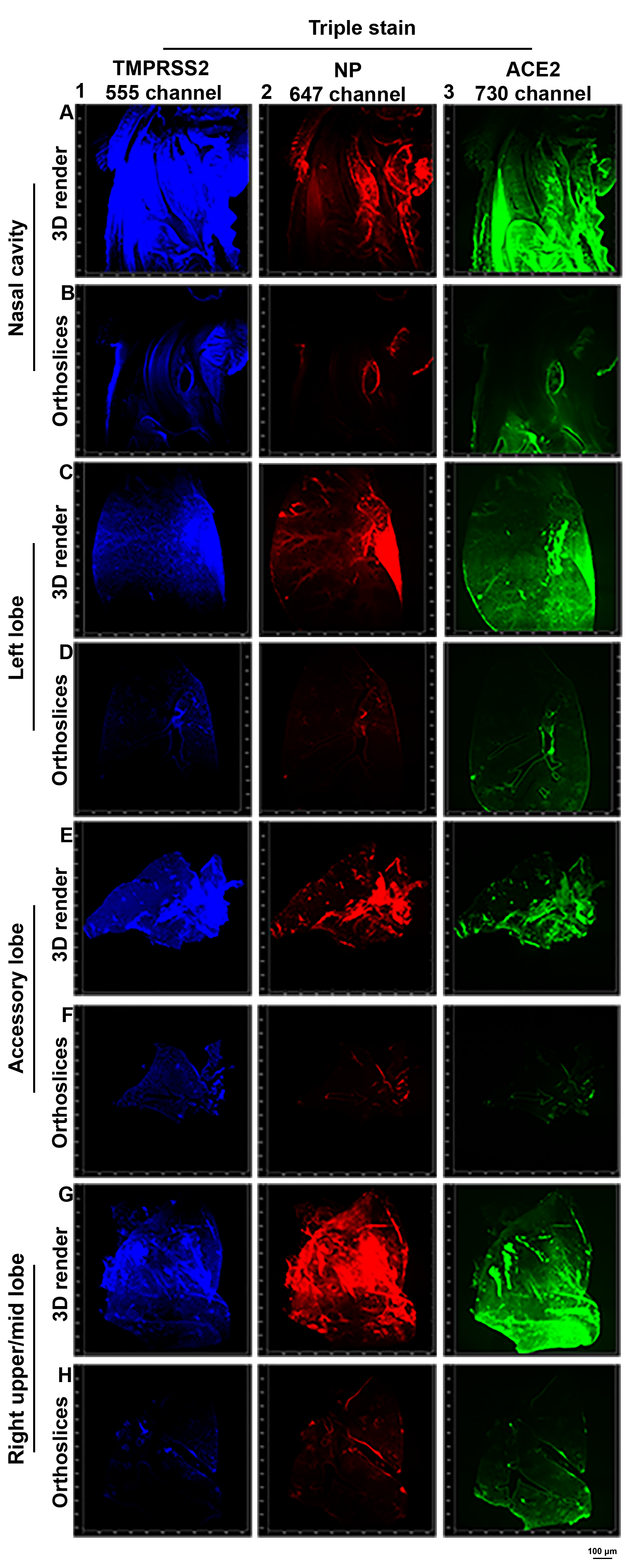

Supplement: S8 Fig — TMPRSS2 imaged in the 555 channel, NP in the 647 channel and ACE2 in the 730 channel. Merges of all channels shown in red (ACE2), green (NP) and blue (TMPRSS2). (A/B) ACE2 and TMPRSS2 signal in the nasal cavity with SARS-CoV-2 infection overlap. 0.63 zoom, voxel resolution X,Y,Z: 4.79 μm, 4.79 μm, 5 μm. (C/D) In the left lobe TMPRSS2 signal is seen in the upper portions of the lung lobe. ACE2 signal mostly lower in the lung lobe. SARS-CoV-2 infection is transposing in regions where both ACE2 and TMPRSS2 signal is present. (E/F) In the accessory lobe fluorescence signal of TMPRSS2, NP and ACE2 overlap. (G/H) The right upper/mid lobe contains TMPRSS2, ACE2 and NP signal superimpose. Regions with SARS-CoV-2 infection either show high TMPRSS2 and low ACE2 signal or low TMPRSS2 and high ACE2. (TIF) [file ppat.1010340.s008.tif]
